# High-Resolution Metagenomics of Human Gut Microbiota Generated by Nanopore and Illumina Hybrid Metagenome Assembly

**DOI:** 10.3389/fmicb.2022.801587

**Published:** 2022-05-12

**Authors:** Lianwei Ye, Ning Dong, Wenguang Xiong, Jun Li, Runsheng Li, Heng Heng, Edward Wai Chi Chan, Sheng Chen

**Affiliations:** ^1^Department of Infectious Diseases and Public Health, Jockey Club College of Veterinary Medicine and Life Sciences, City University of Hong Kong, Kowloon, Hong Kong SAR, China; ^2^College of Veterinary Medicine, South China Agricultural University, Guangzhou, China; ^3^State Key Laboratory of Chemical Biology and Drug Discovery, Department of Applied Biology and Chemical Technology, The Hong Kong Polytechnic University, Hung Hom, Hong Kong SAR, China; ^4^Hong Kong Branch of the Southern Marine Science and Engineering Guangdong Laboratory, Guangzhou, China

**Keywords:** human metagenome, Illumina, nanopore, hybrid assembly, high resolution

## Abstract

Metagenome assembly is a core yet methodologically challenging step for taxonomic classification and functional annotation of a microbiome. This study aims to generate the high-resolution human gut metagenome using both Illumina and Nanopore platforms. Assembly was achieved using four assemblers, including Flye (Nanopore), metaSPAdes (Illumina), hybridSPAdes (Illumina and Nanopore), and OPERA-MS (Illumina and Nanopore). Hybrid metagenome assembly was shown to generate contigs with almost same sizes comparable to those produced using Illumina reads alone, but was more contiguous, informative, and longer compared with those assembled with Illumina reads only. In addition, hybrid metagenome assembly enables us to obtain complete plasmid sequences and much more AMR gene-encoding contigs than the Illumina method. Most importantly, using our workflow, 58 novel high-quality metagenome bins were obtained from four assembly algorithms, particularly hybrid assembly (47/58), although metaSPAdes could provide 11 high-quality bins independently. Among them, 29 bins were currently uncultured bacterial metagenome-assembled genomes. These findings were highly consistent and supported by mock community data tested. In the analysis of biosynthetic gene clusters (BGCs), the number of BGCs in the contigs from hybridSPAdes (241) is higher than that of contigs from metaSPAdes (233). In conclusion, hybrid metagenome assembly could significantly enhance the efficiency of contig assembly, taxonomic binning, and genome construction compared with procedures using Illumina short-read data alone, indicating that nanopore long reads are highly useful in metagenomic applications. This technique could be used to create high-resolution references for future human metagenome studies.

## Introduction

The human gut microbiome is a dynamic and complex microbial ecosystem dominated by bacteria, which interact with the host and directly impact human physiology (Lloyd-Price et al., 2016; Forster et al., 2019; Tan et al., 2021). Classical studies of the gut microbiome were largely dependent on cultivation techniques. However, traditional methods only cultivate 10–30% of gut microbiota (Suau et al., 1999; Tannock, 2001; Sokol and Seksik, 2010). With the rapid development of advanced molecular technologies such as PCR-denaturing gel electrophoresis, it has been demonstrated that the gut microbial ecosystem is more complex than previously thought (Eckburg et al., 2005). In recent years, several next-generation sequencing technologies have been developed (Shendure and Ji, 2008; Fuller et al., 2009; Zhong et al., 2021), thus further facilitating analysis of a large number of microorganism in different environment (Tyson et al., 2004; Venter et al., 2004; Tringe et al., 2005) and human body sites (Ding and Schloss, 2014), including the human gut (Huttenhower et al., 2012; Methé et al., 2012; Tyakht et al., 2013). 16S rRNA gene sequence analysis has been used to study uncultivated gut microbial communities, which focused on the sequence of the conserved 16S rRNA gene present in all microbes (Woese and Fox, 1977; Cole et al., 2006; Oyewusi et al., 2021), and has established a series of novel connections between intestinal microbiota and disease (Cho and Blaser, 2012; Blaser et al., 2013; Ren et al., 2013; Wei et al., 2022). Advent of shotgun metagenome sequencing substantially resolved the technical difficulties associated with taxonomic classification and functional annotation of gut microbiome by offering a way to assess the entire genomic contents (Lloyd-Price et al., 2016; Almeida et al., 2019; Forster et al., 2019; Peterson et al., 2021). With the recent advance in computational approaches, the recovery of metagenome-assembled genomes (MAGs) from highly diverse communities was accessible *via de novo* assembling shotgun metagenomic reads into contig sequences and binning the assembled contigs with similar sequence composition, taxonomic affiliations, and coverage depth (Truong et al., 2015; Parks et al., 2017; Quince et al., 2017; Uritskiy et al., 2018). Metagenome assembly is methodologically more challenging compared with the assembly of single isolates due to the inability to distinguish between closely related community members in both the assembly and binning processes, which limits the accuracy of MAGs-related analyses (Parks et al., 2017; Truong et al., 2017; Forster et al., 2019). Extensive work has been conducted to expand the tree of life by recovering MAGs with high accuracy and completeness, including establishment of reference genome catalogs through cultivation of human gut bacteria, such as Human Microbiome Project (HMP) (Turnbaugh et al., 2007; Integrative et al., 2019) and Human Gastrointestinal Bacteria Genome Collection (HGG) (Forster et al., 2019), increasing the sample size of gut microbiota sequenced with the reference-free and culture-independent approach as well as improving the sequencing output by using long reads generated from third-generation sequencing platforms like Oxford Nanopore Technologies (ONT) and Pacific Biosciences (PacBio) single-molecule real-time (SMRT) sequencing (Bleidorn, 2016; Frank et al., 2016; Mukherjee et al., 2017; Almeida et al., 2019; Bertrand et al., 2019; Pasolli et al., 2019; Zou et al., 2019).

Theoretically, long-read sequencing technologies can overcome many problems associated with those using short reads such as the poor contiguity and ambiguity in metagenome assemblies, but they are more expensive and error-prone (Frank et al., 2016; Wick et al., 2017; Bertrand et al., 2019). The hybrid genome assembly approach that employs reads generated by different platforms is a powerful way to retain the advantage of both short- and long-read sequencing methods and generate larger contigs with fewer misassemblies (Mostovoy et al., 2016; Wick et al., 2017; Ma et al., 2018). It has been successfully applied for the study of human genomes and single bacterial colonies, and there are only a few reports on the use of such a method in microbiome-related studies (Mostovoy et al., 2016; Wick et al., 2017; Jain et al., 2018b; Ma et al., 2018; Li et al., 2021). Frank et al. (2016) reported the enhanced genome construction of the complex microbial community in a commercial biogas reactor by using the combination of Illumina short reads and PacBio long-read circular consensus sequence (CCS) data. Bertrand et al. (2019) recently developed another hybrid metagenome assembler, OPERA-MS, which could accurately generate near-complete genomes from metagenomes with relatively low coverage of long reads (∼9×). Since SMRT sequencing is currently inaccessible to most laboratories because of its high cost and laborious preparation procedure, researchers often work with the portable MinION device available from ONT (Li et al., 2018). Although hybrid approach recovered high-quality MAGs from a complex aquifer system Overholt et al. (2020) and Jin et al. (2022) used MetaBAT2 to assemble 475 high-quality MAGs by HiSeq-PacBio hybrid, there were few reports on the assessment of different hybrid assemblers and Metagenome-assembled genome binning methods.

In this study, we present an application and one novel workflow of combined nanopore MinION long reads and Illumina short reads data in a complex gut microbial community of a healthy man. We compared the contiguity and accuracy of the assemblies of HiSeq X10 short reads, MinION nanopore long reads, and hybrid assemblies from both platforms. A staggered mock community was also constructed to compare the assembly quality from different assembling strategies with ground-truth reference. We demonstrated that, with the advance in data analysis tools, the workflow is feasible for MAG recovery, and that these MAGs can serve as valuable high-resolution references for studying human gut microbiota.

## Materials and Methods

**FIGURE 1:**
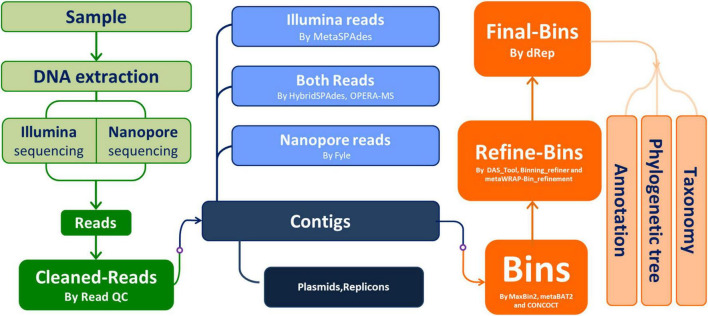
Workflow of this study. The starting sample was the stool sample from a “healthy” Chinese young man.

The major workflow of this study is depicted in Figure 1.

### High-Molecular-Weight DNA Extraction

Metagenomic DNA extraction was carried out using QIAamp DNA Stool Mini Kit (QIAGEN, Valencia, CA, United States), E.Z.N.A. stool DNA kit (Omega Bio-Tek, Norcross, GA, United States), and FastDNA^®^ SPIN Kit (Bio 101, Carlsbad, CA, United States) from the fecal sample of a young healthy man who was 29 years of age, weighing 70 kg, and height 168 cm according to the instructions of the manufacturer. However, E.Z.N.A. Stool DNA Kit and FastDNA SPIN Kit generated a majority of DNA fragments of <5 kb, which were not suitable for nanopore sequencing. DNA was finally extracted using QIAamp DNA with minor modifications. Briefly, we followed the major instructions in the section “Isolation of DNA from stool for pathogen detection” in the second step. After weighing the fresh stool and adding 1 ml InhibitEX Buffer, one sterile 1-ml tip was used to smash the stool and some 0.5 mm sterile glass beads were added to help homogenize the sample. In the fifth step, 2 μl 20 mg/ml RNase A from PureLink Genomic DNA Mini Kit were added; the final volume of sterile water to elute DNA was reduced to 50 μl to obtain DNA with increased concentration. To reduce short DNA fragments, 0.5 × Agencourt AMPure XP beads were used. The quality and quantity of DNA were evaluated by running a 0.5% agarose gel and using the Qubit™ dsDNA BR Assay Kit (Thermo Fisher Scientific Inc., Waltham, MA, United States), respectively. Finally, DNA with high molecular weight (modal size >5 kbp) and sufficient quantity (>20 μg) for sequencing (Supplementary Figure 6) was extracted from the stool sample of a healthy young man without any overt disease as described previously (Aagaard et al., 2013).

### Construction of BMS21 Mock Community

The American Type Culture Collection (ATCC) was used to purchase eight bacterial strains, including *Acinetobacter baumannii* (ATCC 19606), *Enterococcus faecium* (ATCC 29212), *Escherichia coli* (ATCC 25922), *Klebsiella pneumoniae* (ATCC 13883), *Lactobacillus casei* (ATCC 393), *Pseudomonas aeruginosa* (ATCC 27853), *Pseudomonas putida* (ATCC 12633), and *Staphylococcus aureus* (ATCC 29213). A total of 13 other strains belonging to different species, including *Enterobacter asburiae*, *Hafnia alvei*, *Serratia liquefaciens*, *Providencia rettgeri*, *Providencia heimbachae*, *E. coli*, *Ideonella dechloratans*, *Morganella morganii*, *Escherichia cloacae*, *Vibrio vulnificus*, *Streptococcus faecalis*, and *Lactobacillus* spp. isolated from human feces, pig feces, yogurt, and shrimp samples, were stock strains from our lab. DNA extraction was carried out using the PureLink™ Genomic DNA Mini Kit (Invitrogen, Carlsbad, CA, United States) according to the instructions of the manufacturer. Integrity of extracted DNA was inspected on 0.5% agarose gel. DNA concentration was determined by Qubit dsDNA BR assay. A staggered mock community, BMS21, was constructed by pooling DNA for the 21 strains in different abundance levels varying from 0.1 to 30% (Supplementary Table 12). DNA of individual isolates, the BMS21 mock community, and the human metagenome were also subjected to quality and quantity evaluation with the Agilent 2100 Bioanalyzer (Agilent Technologies, Santa Clara, CA, United States). The comparative assessment of BMS21 was carried using AMBER (Meyer et al., 2018) which provides commonly used metrics for assessing the quality of metagenome binnings on benchmark datasets.

### Illumina and Nanopore MinION Sequencing of Metagenomics DNA Sample

DNA of individual isolates, the mock community, and the human metagenome were subjected to both Illumina short-read and nanopore long-read sequencing. Illumina paired-end libraries were prepared by the focused acoustic shearing method with the NEBNext Ultra DNA Library Prep Kit and the Multiplex Oligos Kit for Illumina (NEB) (Li et al., 2018). The libraries were quantified by employing quantitative PCR with P5-P7 primers, and were pooled together and sequenced in the HiSeq X10 platform according to the protocol of the manufacturer (Illumina, San Diego, CA, United States). After read trimming and removal of the human reads, a total of 26 Gb 2 × 150 bp pair-end sequencing data was generated by the Illumina HiSeq X10 apparatus. Libraries of nanopore long-read sequencing were prepared with the Rapid Barcoding Sequencing Kit (SQK-RBK004) and flowcell R9.4 according to the protocols of the manufacturer. The sequencing run was stopped after 8 h, and the flow cell was washed by a Wash Kit (EXP-WSH002) (Li et al., 2018).

### Metagenome Assembly, Contiguity Estimation, and Metagenome Binning

Illumina raw reads were trimmed and sequences belonging to the human genome were removed using the READ_QC module in metaWRAP version 1.1.5 (Uritskiy et al., 2018). Nanopore reads were basecalled and debarcoded with guppy version 3.1. Nanopore reads were assembled into contigs with Flye version 2.9 (Kolmogorov et al., 2020) using a genome size of 100 Mbp, and the Illumina reads were assembled using metaSPAdes version 3.15.3 using default parameters (Koren et al., 2017; Nurk et al., 2017). Hybrid assembly of reads from both platforms was conducted using hybridSPAdes version 3.15.3 (Bankevich et al., 2012) and OPERA-MS (Bertrand et al., 2018), respectively. MetaQUAST version 5.0.2 was used to evaluate all metagenome assemblies and obtain statistics including N50, genes assembled and misassembly errors (Mikheenko et al., 2015). Specifically, misassemblies is the number of positions in the assembled contigs where the left flanking sequence aligns over 1 kb away from the right flanking sequence on the reference or they overlap on more than 1 kbp or flanking sequences align on different strands or different chromosomes. The PlasFlow (Krawczyk et al., 2018) was used to classify the contigs generated by four assemblers. Binning of metagenomic contigs was conducted using MaxBin 2.0 (Wu et al., 2015), MetaBAT2 (Kang et al., 2015), and CONCOCT (Alneberg et al., 2014) embedded in metaWRAP version 1.1.5 using default parameters (Uritskiy et al., 2018). A refinement step was then performed using the bin_refinement module from MetaWRAP to combine and improve the results generated by the three binners, the cutoff value of genome completeness was set to 50%, and that of contamination was 10% (Uritskiy et al., 2018). Self-mapping was conducted with Bowtie2 (Langmead and Salzberg, 2012) and SAMtools (Li et al., 2009). The running times/memory consumption of the assemblers are described in the Supplementary Table 13.

### Dereplication and Characterization of the Metagenome-Assembled Bins

The refined bins generated for contigs from each metagenome assembly methods were subsequently dereplicated with dRep version 2.3.2 to extract the MAGs displaying the best quality and representing individual metagenomic species (Olm et al., 2017). The lineage, completeness, and contamination of the recovered MAG were estimated using CheckM version 1.1.3 (Parks et al., 2015) with lineage-specific marker genes. The GTDB-Tk was used to identify the classification of bins. Average nucleotide identity (ANI) of the bins with related genomes was calculated using OrthoANI (Lee et al., 2016). SNP calculation was conducted using snippy version 3.2 (Seemann, 2015).

### Assignment of Metagenome-Assembled Genomes to Reference Databases

Three reference databases were used to classify the set of MAGs in our study recovered from the human gut microbiome, namely, HR, RefSeq, and a collection of MAGs from public datasets. HR comprised a total of 2,110 high-quality genomes (>90% completeness and <5% contamination) retrieved from both the HMP catalog^1^ and the HGG (Forster et al., 2019). From the RefSeq database, we used all the complete bacterial genomes and chromosome available (*n* = 30,057). Finally, we surveyed 92,143 MAGS database (Almeida et al., 2019)^2^. For each database, FastANI was used to calculate the whole-genome ANI (Jain et al., 2018a). Subsequently, each MAG and its closest relative compared their aligned sequence fragments. These unclassified MAGs were clustered into phylum level using GTDB-Tk (Chaumeil et al., 2020).

### Phylogeny of the Metagenome-Assembled Bins

Using specI version 1.0, forty universal core marker genes from each genome bin were extracted (Mende et al., 2013). Phylogenetic trees were built by concatenating and aligning the marker genes with MUSCLE version 3.8.31 (Edgar, 2004). Marker genes absent only from specific genomes were kept in the alignment as missing data. Maximum-likelihood trees were constructed using RAxML version 8.2.11 with option -m PROTGAMMAAUTO. All phylogenetic trees were visualized and modified in iTOL (Stamatakis, 2014; Letunic and Bork, 2016).

### Analysis of Plasmids, Mobile Elements, and Antimicrobial Resistance Genes

Plasmid sequences were identified by looking for plasmid replicons using PlasmidFinder 2.1 (Carattoli et al., 2014) and PlasFlow (Krawczyk et al., 2018). Completeness of the plasmids was identified by inspecting the similarity of plasmid sequences at both ends. Acquired antibiotic resistance genes were identified with ResFinder 2.1 using the genome assemblies as input (Zankari et al., 2012). Antibiotic resistance genes with >98% of the sequence aligning to the contig with an identity >99% were selected for further analysis. Insertion sequences were identified using ISfinder (Siguier et al., 2006). Plasmids were annotated with the RAST server (Overbeek et al., 2013). Map of plasmids was plotted using BRIG (Alikhan et al., 2011).

## Results

### Hybrid Metagenome Assembly Improves Assembly Quality

**FIGURE 2:**
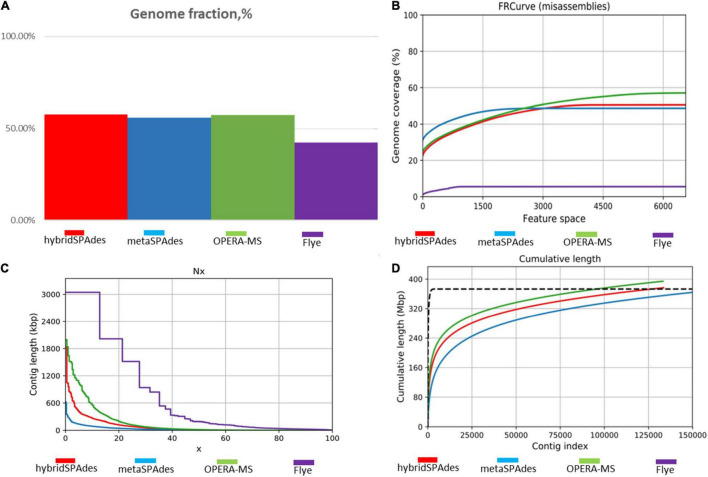
Comparison of the healthy human gut metagenome assembly statistics from the four different assembly methods. **(A)** Genome fraction depicted by different methods. Genome fraction is the percentage of aligned bases in the reference genome. A base in the reference genome is aligned if there is at least one contig with at least one alignment to this base. Contigs from repetitive regions may map to multiple places, and thus may be counted multiple times. **(B)** Feature-response misassembly curve. *Y* is the total number of aligned bases divided by the reference length, in the contigs having the total number of misassemblies at most *X*. FRCurve definition: given any such set of features, the response (quality) of the assembler output is then analyzed as a function of the maximum number of possible errors (features) allowed in the contigs. **(C)** Percentage distribution (*X*-axis) of contig length (*Y*-axis) with the four methods. **(D)** Cumulative number of assembled nucleotides in contigs of different lengths. Each line corresponds to a different assembly program (hybridSPAdes, metaSPAdes, OPERA-MS, and Flye).

**FIGURE 3:**
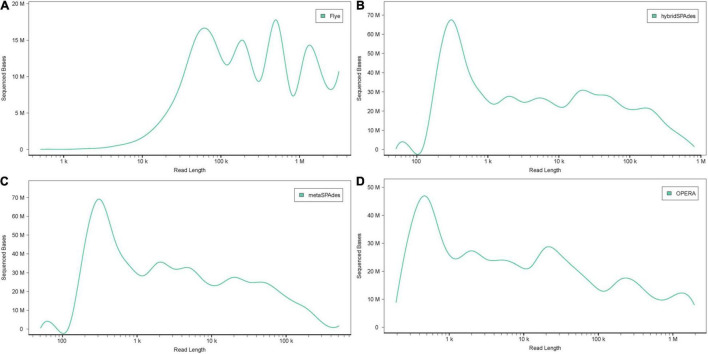
Accumulative distribution of contig length with the four methods: **(A)** Flye, **(B)** hybridSPAdes, **(C)** metaSPAdes, and **(D)** OPERA-MS. The *X* and *Y* axis represent the length (bp) and number of the contigs, respectively.

**FIGURE 4:**
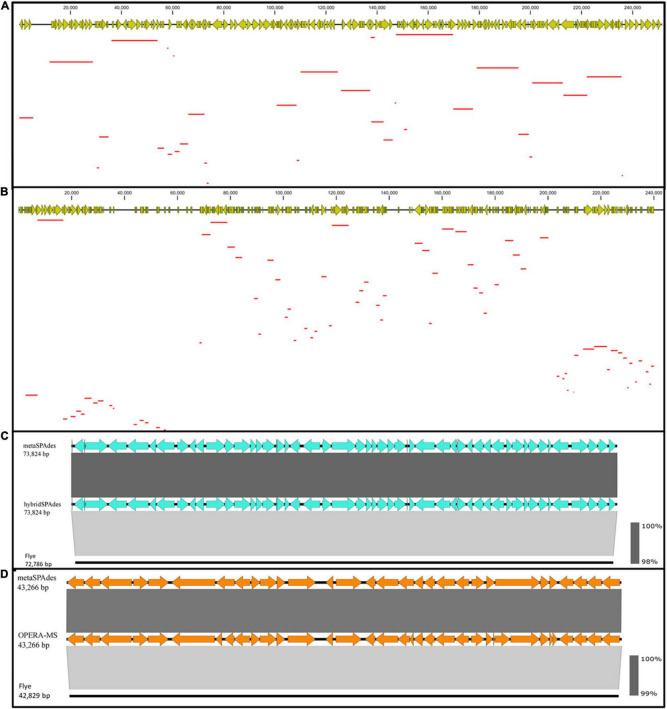
Contiguity and accuracy of assembly with four different assembly algorithms. BLASTN of a 242,790 bp **(A)** and 242,845 bp **(B)** contigs assembled using hybrid assembly methods, hybridSPAdes **(A)**, and OPERA-MS **(B)** against the metaSPAdes assembly with Illumina reads alone. This result indicated that the hybrid assembly generates more contiguous contigs. Linear alignment of contigs assembled using hybridSPAdes [**(C)** ∼73,824 bp] and OPERA-MS [**(D)** ∼43,266 bp] with assemblies constructed using Flye and metaSPAdes. The results indicated that the hybrid assembly generated contigs with high accuracy. The red lines represent Illumina contigs matched to hybrid assembled contig.

Two nanopore MinION flow cells generated a total of 1,205,055 base-called reads containing 5.4 gigabases, with a read N50 (read length refers to reads equal to or longer than this length in at least half of the total bases) of 9,521 bp and a maximum read length of 85,079 bp (Supplementary Figure 1). To analyze data generated by the two different sequencing platforms, multiple metagenome assembly algorithms were used. The metaSPAdes version 3.15.3 program was used to assemble the HiSeq X10 reads and generate 150,855 contigs with a size larger than 0.5 kb, a maximum length of 595,004 bp, and an average contig length of 2,400 bp. Flye version 2.9 was used to assemble the nanopore MinION reads and generated 1,968 contigs (>0.5 kb) averaging 74,028 bp, with the maximum contig length of 2,947,413 bp. Hybrid metagenome assembly with both short- and long-sequencing reads was conducted with two software, hybridSPAdes version 3.15.3 and the recently developed hybrid metagenomic assembler OPERA-MS. Assembly with hybridSPAdes produced 131,093 contigs (>0.5 kb) with an average size of 2,854 bp and a maximum contig length of 807,998 bp. OPERA-MS assembly generated 134,680 contigs (>0.5 kb) with an average length of 2,813 bp and a maximum contig length of 3,008,007 bp. The N50 of contigs (>500) assembled from metaSPAdes, Flye, hybridSPAdes, and OPERA-MS were 6,048, 227,485, 11,867, and 12,770, respectively. Numbers of contigs longer than 500 kb that were assembled by Flye, metaSPAdes, hybridSPAdes, and OPERA-MS were 48, 2, 11, and 39, respectively (Table 1), suggesting that the use of nanopore long-reads improved the assembly contiguity of human metagenome. Hybrid assemblies using OPERA-MS and hybridSPAdes generated metagenome sizes that were similar to those generated by the short-read-only assembly using metaSPAdes, 378, 374, and 362 Mb, respectively. Such sizes were around 2.5-fold the total assembly size from Flye assembly (142 Mb). The self-mapping rates of Illumina short pair-end reads to four assemblies were 45.5% (Flye), 79.0% (OPERA-MS), 94.2% (metaSPAdes), and 95.5% (hybridSPAdes), respectively. Comparison of the assembly statistics of the four assemblies showed that assembly with the nanopore reads alone generated the longest contigs, but the total assembly size and the assembly accuracy were much lower than those generated by the other three assembly methods involving Illumina short reads (Figures 2C, 3, Table 1, and Supplementary Table 1). Considering the high-cost, high error rate, low-throughput of long-read sequencing and taking datasets generated in this study into account (Figure 4), Illumina short-read sequencing was considered essential for improving the accuracy and completeness of metagenome assembly.

**TABLE 1:** Assembly statistics of different assembly algorithms for the healthy human gut microbiome.

Reads	Assembly method	No. of contigs (>500 bp)	No. of contigs (>1 Mb)	No. of contigs (>500 kb)	No. of contigs (>100 kb)	Total assembly size (Mb)*	Mean (bp)	Median (bp)	Max (bp)
Nanopore	Flye v2.9	1968	20	48	267	145	74,028	28,893	2,947,413
HiSeq X10	metaSPAdes v3.15.3	150,854	0	2	197	362	2,400	897	595,004
Hybrid	hybridSPAdes v3.15.3	131,093	0	11	342	374	2,854	866	807,998
Hybrid	OPERA-MS	134680	15	39	293	378	2,813	842	1,996,746

*Contigs less than 500 bp were not calculated.

To demonstrate the contiguity of hybrid assembly, alignment between contigs generated by OPERA-MS/hybridSPAdes and metaSPAdes was conducted. A total of 32 contigs assembled with metaSPAdes were aligned to a 242,790 bp contig generated by hybridSPAdes. Such contig was found to encode 185 ORFs whose size ranged from 248 bp to 22,228 bp when generated by hybridSPAdes. Among these 32 contigs, only 6 are longer than 10,000 bp (Figure 4A). A BLAST search in the NCBI Nucleotide collection (nr/nt) database indicated that it was 81.84% identical to the *Sutterella* sp. KGMB03119 chromosome sequence (accession: CP040882.1) at 21% coverage, indicating that this contig may originate from an unknown genome. Consistently, a total of 77 contigs ranging from 630 bp to 9,772 bp assembled with metaSPAdes were aligned to a 242,845 bp OPERA-MS-generated contig that comprised 328 genes. The 77 contigs comprised a total of 201 genes, with the majority being less than 5 kb. A BLAST search in the NCBI database suggested it was a novel sequence that was 94.50% identical to the chromosomal sequence of *E. coli* strain 602354 (accession: CP025847.1) at 29% coverage (Figure 4B). The sequence alignment results indicated that a hybrid metagenome assembly contains more contiguous and informative, as well as longer contigs compared to those assembled with Illumina reads only. Assembly with the Illumina reads alone generated contigs with the highest accuracy, but the low contiguity of such contigs limits their application potential. Hybrid metagenome assembly with both nanopore long- and Illumina short reads could be an effective approach that integrates the strength of both sequencing platforms. The two currently available hybrid metagenome assembly algorithms, OPERA-MS and hybridSPAdes, enabled high-quality assemblies with low-coverage nanopore long reads and fragmented Illumina short reads, with the former performing better on the contiguity (number of long contigs which are >500 kb, Table 1). The difference in performance could be due to the difference in the discrimination and assembly principles of the two algorithms. HybridSPAdes conducts hybrid assembly by mapping the third-generation long reads to the assembly of second-generation short reads, and OPERA-MS integrates a novel assembly-based metagenome clustering technique with an exact scaffolding algorithm that can efficiently assemble repeat-rich sequences (Antipov et al., 2015; Bertrand et al., 2019).

### Construction of Near-Complete and High-Fidelity Metagenome Bins With Hybrid Assembly Algorithms

**FIGURE 5:**
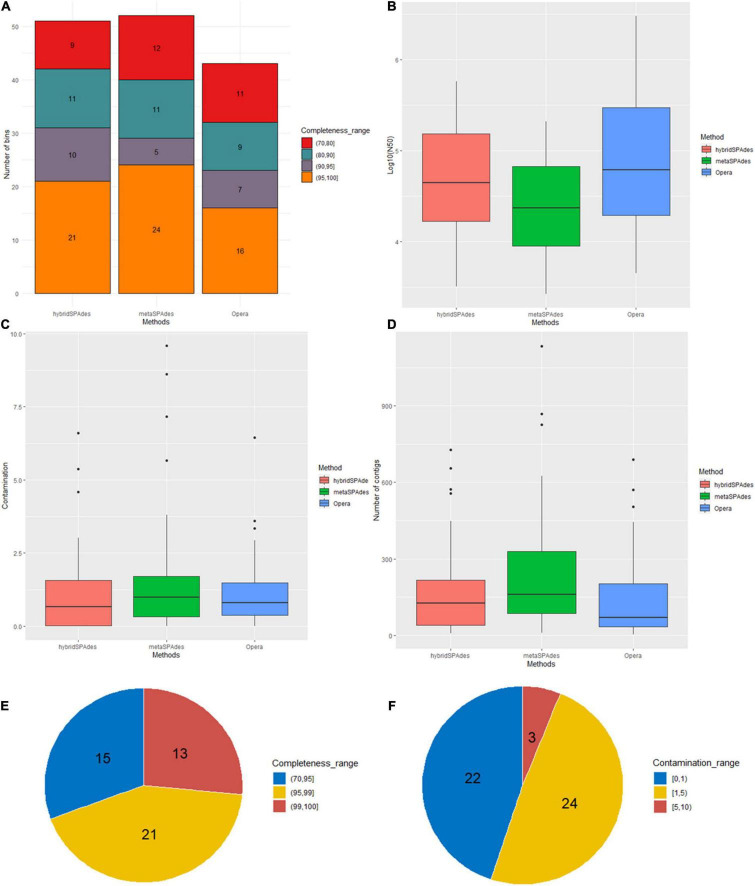
Binning statistics of genome assembly with different algorithms (metaSPAdes, hybridSPAdes, and OPERA-MS). **(A)** Number of genome bins with different completeness (>95%, 90–95%, and 70–95%). **(B)** log10 N50 of the genome bins. **(C)** Contamination percentages (%) of the genome bins. **(D)** Distribution of number of contigs in each bin. Distribution of completeness **(E)** and contamination **(F)** of metagenome bins after dereplication.

Binning of the assembled metagenome sequences generated with the four algorithms (metaSPAdes, hybridSPAdes, and OPERA-MS) was conducted using three different software (MaxBin2, MetaBAT2, and concoct), resulting in generation of primary metagenome bins (Table 2, Supplementary File 1, and Supplementary Table 2). The metaSPAdes assembler generated 349 bins. The number of bins with less than 100 contigs was 142. In these bins, only 38 were at the completeness of >50% and <10% contamination, and the number of high quality (completeness >95% and contamination <3%) were 19. A total of 199 bins were generated by OPERA-MS assembler and the number of bins with less 100 contigs was 81, 21% (42) of which exhibited completeness of >50% and <10% contamination and 9.5% (19) were high-quality bins. A total of 179 of the 373 bins assembled by hybridSPAdes with less 100 contigs were at completeness of >50% and contamination. The number of high-quality (completeness >95% and contamination <3%) bins was 28 (7.4%). The N50 of the contigs in each bin were 1,999–573,607 (metaSPAdes), 2,728–3,008,073 (OPERA-MS), and 1,024–596,353 (hybridSPAdes), respectively (Supplementary File 1). Comparison with bins from metaSPAdes, bins assembled by Illumina and nanopore reads show higher quality and better quantity. The primary bins were refined with DAS_Tool and finalized using metaWRAP-Bin_refinement using a completeness cutoff of 50% and contamination cutoff of 10%. A total of 156 bins were obtained from different metagenome assemblies, including 52, 51, and 53 from, metaSPAdes, hybridSPAdes, and OPERA-MS, respectively (Figure 5A). The number of bins and the corresponding bin completeness generated by metaSPAdes and hybridSPAdes are similar, which are slightly more than that recorded in hybrid assembly using OPERA-MS, but the number of contigs in each bin decreased in bins of hybrid assembled contigs compared with contigs assembled with the Illumina reads alone (Figure 5D and Supplementary File 1). The N50 of the contigs in each bin were, respectively, 2,642–209,184 bp (metaSPAdes), 3,163–578,001 bp (hybridSPAdes), and 4,467–3,008,073 bp (OPERA-MS) (Figure 5B). The numbers of bins with less than 100 contigs are 17 (32.0%), 22 (43.1%), and 24 (55.8%) for assembly with metaSPAdes, hybridSPAdes, and OPERA-MS, respectively. These data indicated the efficiency of metagenome binning with hybrid genome assemblies was enhanced by increasing contig length and decreasing number of contigs in each bin without introducing more contamination.

**TABLE 2:** Summary of primary bins and refined bins generated.

	Primary bins				metaWRAP refine-bin			
		
	No. of Primary-bins	No. of contig <100	Comp.*^a^* >50 and cont.*^b^* <10%	Comp. >95 and cont. <3%	N50	No. refine-bins	N50	No. of contig <100
metaSPAdes	349	142	38	19	1,999–573,607	52	2,642–209,184	17
HybridSPAdes	373	277	179	28	2,728–3,008,073	51	3,163–578,001	22
OPERA-MS	199	81	42	19	1,024–596,353	53	4,467–3,008,073	23

*^a^Completeness.*

*^b^Contamination.*

### Metagenome-Assembled Genomes in the Human Gut Microbiome

Comparison and dereplication of metagenome bins generated from the four assemblies using dRep resulted in the generation of a total of 58 bins, among which 11 was from assembly with Illumina reads alone, and the remaining 47 were from binning of hybrid assemblies (14 from OPERA-MS, 33 from hybridSPAdes, Supplementary Figure 2). The percentage of genome completeness ranged from 71.3 to 100% and that of the contamination level was between 0 and 6.5% (Figure 5 and Table 3). The number of contigs in the 58 bins ranged from 7 to 689, with 37 bins (63.8%) containing contigs less than 200 contigs (Table 2). Of note, the five bins with lowest number of contigs, which ranged from 7 to 30 contigs, were generated using contigs from hybridSPAdes (4) and metaSPAdes (1). Compared with these bins, the number of contigs in metaSPAdes-assembled bins is mostly more than 50 (10/11) and there are eight bins that contain more than 90% of the contigs that were less than 100 kb. The completeness of the seven bins was >80% except for one with 77.2% completeness and contamination was <3%. Among them, three bins with less than 10 contigs at the completeness of >97% and <2% contamination were identified and the largest contigs in these three bins were all more than 0.8 Mb, indicating that hybrid metagenome assemblies prompt the generation of near-complete and high-fidelity metagenome bins.

**TABLE 3:** General genomic features of all bins reconstructed from dereplication of metagenome assembly with different algorithms.

Bin ID	No. of contig	Length (bp)	NGA50 (bp)	No. of tRNA	No. of tmRNA	No. of protein coding genes	GC content (%)	No. of rRNA	Comp.^a^ (%)	Cont.^b^ (%)	No. of gene annotated by COG
hySP11	269	1,769,144	10,331	27	1	1,599	0.599	1	78.9	6.598	578
hySPA12	74	3,112,166	88,795	36	0	2,389	0.565	2	97.98	0.335	829
hySPA13	189	2,663,870	27,772	30	1	2,269	0.36	0	97.98	0.167	807
hySPA14	39	4,005,050	156,162	43	1	2,435	0.482	3	99.23	0	810
hySP18	175	2,288,156	13,249	44	1	2,189	0.491	0	91.77	0.843	826
hySPA20	655	2,495,082	20,138	26	0	2,247	0.382	0	87.07	1.067	812
hySPA21	181	4,164,099	5,175	12	1	1,853	0.426	0	86.25	0.716	638
hySPA22	573	2,504,650	44,101	43	1	2,533	0.507	0	83.9	1.901	754
hydSPA25	126	3,292,018	5,617	26	0	2,117	0.415	1	84.76	0.019	761
hydSPA27	219	2,376,518	40,640	32	1	2,655	0.563	0	97.61	0.68	936
hySPA29	154	2,167,563	17,597	24	1	2,079	0.342	0	93.68	1.86	760
hydSPA3	208	1,763,270	26,437	29	1	2,001	0.395	0	94.41	2.469	701
hydSPA31	14	2,618,360	15,581	36	1	2,377	0.461	1	100	1.197	1,117
hySPA32	149	2,183,825	342,806	56	1	2,431	0.598	5	95.96	0.806	867
hySPA34	215	4,831,208	23,761	51	1	2,007	0.581	0	96.71	1.091	716
hySPA35	449	2,306,057	44,008	61	0	6,929	0.423	11	80.65	2.013	2,807
hySPA36	191	2,511,299	6,595	24	1	1,897	0.599	0	90.49	0.48	677
hySPA38	39	2,727,000	22,674	29	1	1,733	0.614	0	98.75	0.621	588
hySPA39	35	2,975,866	167,826	66	1	2,457	0.413	8	99.51	0	922
hySPA4	29	2,522,937	153,428	29	1	2,637	0.576	0	98.65	0	921
hySPA4	29	2,522,937	151,635	46	1	2,111	0.576	2	98.65	0	725
hySPA41	138	1,924,037	23,085	32	0	1,913	0.482	0	95.13	0.559	693
hySPA43	61	4,228,141	114,127	49	1	2,823	0.432	1	98.92	0	917
hySPA44	205	1,810,309	15,099	27	0	1,725	0.613	1	87.63	3.02	600
hySP46	391	2,211,788	7,930	9	0	2,209	0.289	0	88.52	0	796
hySP47	59	2,309,764	70,537	44	1	2,083	0.582	3	94.47	0	741
hySPA48	116	4,882,463	183,297	37	1	2,639	0.422	2	82.44	2.944	820
hySPA5	250	4,227,969	26,976	41	0	6,183	0.511	6	92.44	0.453	2,585
hySPA50	85	2,430,178	53,327	41	1	1,983	0.41	4	98.99	1.006	680
hySPA51	445	2,294,131	6,300	14	0	2,115	0.469	0	78.61	5.37	771
hySPA6	183	2,552,049	21,470	23	0	2,273	0.504	0	90.31	0.41	811
hySPA7	7	1,834,142	578,001	47	1	1,823	0.366	7	98.65	0	642
hySPA8	411	1,976,503	6,078	27	0	1,887	0.343	0	79.09	2.322	664
SPAd1	87	4,165,019	103,305	37	1	2,721	0.461	0	96.42	0.247	862
mSPA14	125	1,902,876	78,191	38	1	1,765	0.591	0	84.6	5.668	625
mSPA15	75	3,257,610	7,534	35	1	2,441	0.365	0	98.65	0	817
mSPA.20	363	2,188,068	38,734	17	0	1,739	0.448	0	77.24	0.393	605
mSPA21	132	2,878,203	115,100	40	1	1,999	0.596	1	99.18	0.24	664
mSPA36	25	2,146,454	23,934	43	1	1,731	0.545	0	99.51	0.961	606
mSPA38	243	3,152,086	25,918	31	1	2,617	0.419	0	91.69	1.011	935
mSPA40	236	3,782,419	57,354	33	0	2,549	0.456	0	91.74	1.794	837
mSPA44	80	3,137,080	119,525	29	1	2,731	0.417	0	97.58	0.483	941
mSPA.46	51	3,299,494	44,325	43	1	2,153	0.454	0	98.51	0.277	704
mSPA47	94	2,771,004	21,825	10	0	2,023	0.455	0	79.11	0	678
Op1	200	2,317,819	374,219	32	1	2,075	0.563	1	96.54	0.68	737
Op2	202	2,492,862	844,209	49	1	2,273	0.599	10	89.5	0.48	824
Op24	504	2,071,756	55,129	68	1	3,279	0.595	15	71.31	1.469	1,043
Op26	570	1,872,933	14,003	59	1	2,467	0.503	2	71.57	3.601	864
Op29	71	2,743,675	856,673	42	1	2,295	0.487	9	99.05	0.632	1,080
Op3	689	2,530,979	306,231	49	0	2,475	0.286	11	75.43	0	823
Op30	115	3,184,703	204,798	50	1	2,159	0.418	2	92.63	0.672	756
Op31	71	2,135,893	1,541,592	38	0	1,837	0.406	8	98.99	1.006	641
Op34	40	3,825,506	2,482,255	39	1	2,873	0.482	8	98.51	0	880
Op35	232	1,706,406	609,88	58	0	3,031	0.395	17	84.36	3.337	941
Op39	205	8,306,390	296,992	28	1	1,675	0.415	6	74.29	6.45	594
Op41	170	2,138,655	231,829	51	1	2,895	0.599	7	95.16	0.806	1,038
Op42	181	2,133,167	562,214	39	0	6,821	0.343	17	89.5	1.069	2,864
Op43	32	2,582,353		42	1	1,839	0.462	4	99.85	1.197	596

*^a^Completeness.*

*^b^Contamination. Op, OPERA-MS; mSPA, metaSPAdes; hySPA, hybridSPAdes.*

**FIGURE 6:**
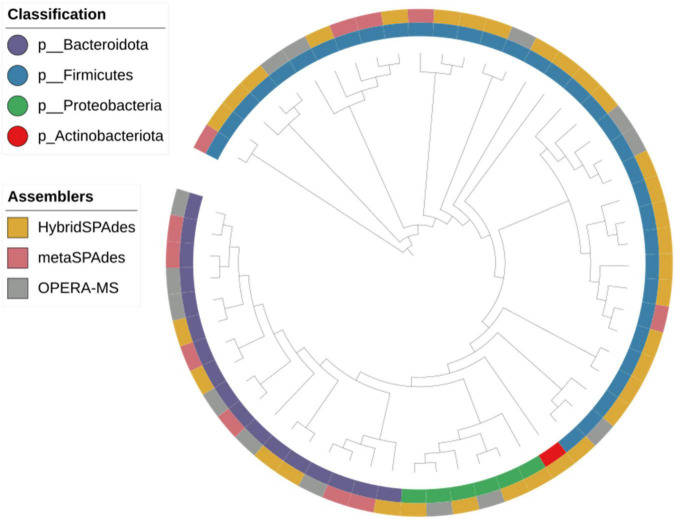
Phylogeny of the genome bins reconstructed from dereplication of metagenome assembly with different algorithms. Phylum of the strains and assemblers are plotted in the figure.

**FIGURE 7:**
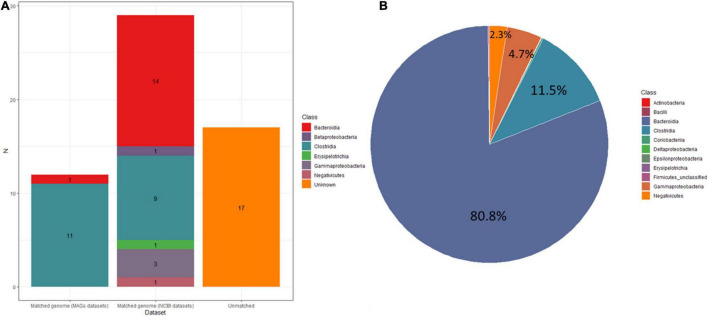
**(A)** Stacked bar plots showing the number of MAGs matched in UMGS and HR datasets or unknown. **(B)** Pie figure showing relative abundance of the gut microbiota. The different colors represent different bacteria at class level.

To determine how many of the MAGs belong to species that have been isolated from pure bacterial cultures (i.e., isolate genomes), we attempted to assign these MAGs to all bacteria references of NCBI datasets (RefSeq database) and 2,110 isolate genomes (HR database) combined from HMP and HGG (Forster et al., 2019). In addition, we also compared the 58 MAGs to a set of 92,143 MAGs from 11,850 human gut metagenome (Almeida et al., 2019), including 1,952 unclassified bacterial MAGs (UMGs). Of the 58 MAGs, we were able to assign 29 MAGs and 12 MAGs to the HR and UMGs dataset, respectively, using a criterion of at least 60% of aligned fragment (AF) with at least 95% ANI. Among the 29 MAGs, there were two most frequent genomes assigned to the class (Bacteroidia *n* = 14, Clostridia *n* = 9). All are known colonizers of the human gut, confirming that these species are common members of the intestinal microbiota (Figures 6, 7A and Supplementary File 4). In addition, it was consistent with the microbiome abundance obtained from metagenomic analysis (Figure 7B). Meanwhile, twelve MAGs matched to the UMGs dataset were Clostridia (*n* = 11) and Bacteroidia (*n* = 1). However, there still were 17 MAGs that were not matched in these two datasets, while they were clustered by GTDB-Tk into Firmicutes (*n* = 14), Bacteroidota (*n* = 1), Proteobacteria (*n* = 1), and Actinobacteriota (*n* = 1) (Figures 6, 7 and Supplementary File 4). This indicated our workflow has a positive effort in researching unclassified bacterial.

### Plasmids and Antimicrobial Resistance Genes in Human Microbiome

**FIGURE 8:**
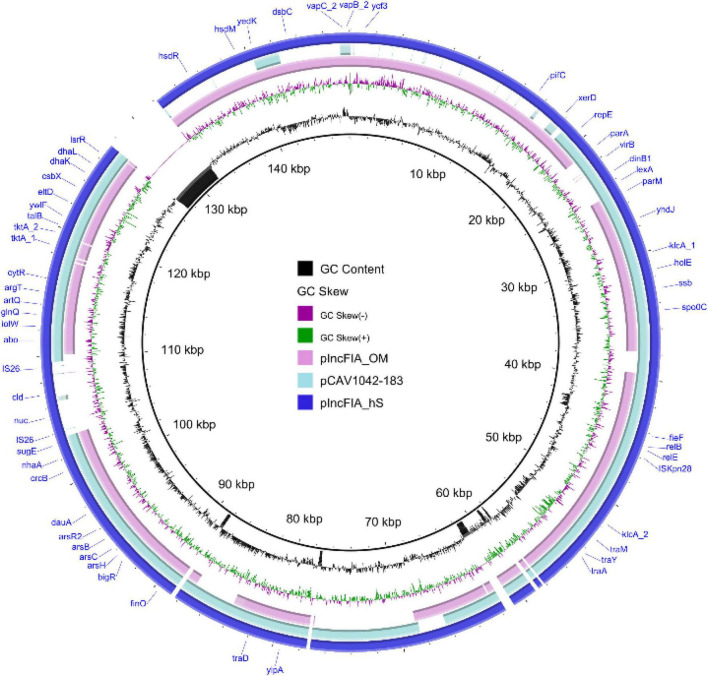
Map of the largest observed new completed circular plasmid sequence using hybrid assembly methods, hybridSPAdes, and OPERA-MS. Plasmids pIncFIA_hS, pIncFIA_OM, and pCAV1042_183 are plotted in the figure using the sequence of pIncFIA_hS as a reference.

Plasmids are major genome contents of bacteria, which normally carry genes that benefit the survival of the organism, such as the antimicrobial resistance genes. Due to the carriage of large numbers of Insertion Sequences in MDR plasmids, short-read Illumina sequencing become challenging in getting complete MDR plasmid sequences. To compare the plasmid contents resolved by different assembly algorithms, contigs carrying the plasmid replicons were extracted. A total of 32.5% (38,570/118,507) and 29.3% (39,433/134,361) contigs generated by hybridSPAdes and OPERA-MS were identified as chromosomal-related contigs that were clustered into the phylum level, respectively. The chromosomal data for contigs from Flye and metaSPAdes assemblers were 79.0% (1556/1968) and 31.2% (47,034/150,470), respectively. The number of plasmid (>10 kbp) assembled by Flye, metaSPAdes, hybridSPAdes, and OPERA-MS were 65, 129, 174, and 164, respectively (Table 4 and Supplementary Table 3). Plasmid replicons identified in assemblies metaSPAdes, hybridSPAdes, and OPERA-MS were highly consistent, with a few replicons identified only in hybrid assemblies (hybridSPAdes and OPERA-MS) (Supplementary File 2). The largest plasmid contig identified in assembly with Flye, metaSPAdes, OPERA-MS, and hybridSPAdes were 145,633, 162,508, 229,251, and 214,848 bp, respectively (Table 4 and Supplementary Table 4). The alignment of the 214,848 bp plasmid and the contigs from metaSPAdes assembly could be seen in Supplementary Figure 3. The top 10 longest plasmids generated by the four programs are also shown in Supplementary Table 4. Contigs (152,484 bp, hybridSPAdes) pIncFIA_hS and (157,875 bp, OPERA-MS) pIncFIA_OM were both complete plasmid sequences that belonged to IncFIA plasmids and shared 99.95% identity at 79% coverage. pIncFIA_hS and pIncFIA_OM were novel plasmids, which exhibited 99.9% identity to plasmid pCAV1042_183 (GenBank accession: CP018670) at 69 and 63% coverage, respectively (Figure 8 and Supplementary Table 4). We identified 5, 17, 27, and 29 different antimicrobial resistance genes with Flye, metaSPAdes, hybridSPAdes, and OPERA-MS assemblies, respectively (Supplementary Table 5). At least 10 genes, including *floR*, *sul1*, and *sul2*, assembled with hybrid algorithms were missing in assembly with single read types. Additionally, contigs carrying AMR genes were identified in 2, 2, 5, and 4 contigs from Flye, metaSPAdes, hybridSPAdes, and OPERA-MS assemblers, respectively (Supplementary File 2). Importantly, hybrid assembly methods (hybridSPAdes and OPERA-MS) enabled us to obtain more contigs/plasmids carrying AMR genes compared to single assembly methods (Flye and metaSPAdes) (Supplementary Figure 4 and Supplementary Table 6). These findings indicated the advantage of hybrid assembly in AMR-related research, including completed plasmid and mobile element sequences.

**TABLE 4:** Summary of chromosomal and plasmids contigs.

	Chromosomal-contig	No. of plasmid (>10 Kbp)	Largest plasmid contig
metaSPAdes	31.20%	129	162,508 bp
HybridSPAdes	32.50%	174	214,848 bp
OPERA-MS	29.30%	164	229,251 bp
Flye	79.00%	65	145,633 bp

### Biosynthetic Gene Cluster Prediction

**FIGURE 9:**
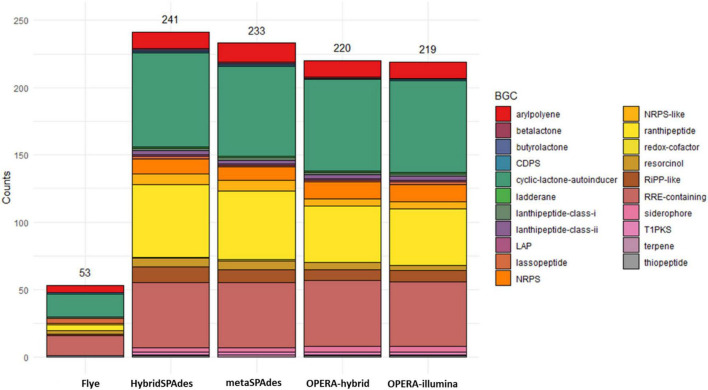
Number of biosynthetic gene clusters (BGCs) predicted by antiSMASH for each draft assembly.

The genome contiguity, completeness, and accuracy have significant effect on gene prediction. Biosynthetic gene clusters (BGCs) are especially influenced by these factors since they are usually found in repetitive regions that are often poorly assembled. AntiSMASH was used to assess the number of clusters found in the draft assemblies in comparison to the reference metagenome with the aim of evaluating BGC prediction on metagenomic assemblies (Figure 9). The number of BGCs recovered by hybrid assemblers (OPERA-MS and HybridSPAdes) is higher than that of metaSPAdes. HybridSPAdes assembler improves the number of BGCs recovered. Meanwhile, the analysis of two MAGs’ BGC shows that one MAG assembled by HybridSPAdes carried one more BGC cluster named resorcinol compared with (99.7% similarity) one MAG assembled by metaSPAdes (Supplementary Figure 7). These findings indicated that the higher completeness MAGs assembled by hybrid assembler have positive effect on the downstream analysis.

### The BMS21 Mock Community Datasets

Mock community standards are essential for the validation of metagenome-related bioinformatics approaches, and the development of genomics methods (Nicholls et al., 2019). To validate the results of human gut metagenome, we constructed a mock community named BMS21 from a low-complexity microbial community with 21 bacterial genomes (accounted for 0.01–30%) (Supplementary Table 12), for which the ground truth was known, and evaluated its assembly datasets. A total of 61 Gb 2 × 150 bp high-quality pair-end Illumina sequencing data and 18.6 Gb base-called nanopore reads were generated for the BMS21 mock community. Assembly with algorithms Flye, metaSPAdes, hybridSPAdes, and OPERA-MS resulted in generation of a metagenome size of 71, 94, 95, and 93 Mb with N50 of 4,185,707, 93,165, 209,776, and 385,369 bp, respectively (Supplementary Table 7). The numbers of contigs assembled with each method were 229, 2,999, 1,812, and 2,521, with the size of the longest contig being 6,834,171, 670,411, 2,247,228, and 6,176,973 bp, respectively. The BMS21 benchmark results are shown in Supplementary File 3. Flye had the highest NGA50s for 18 bacterial genomes. The number of misassemblies and misassembled contigs length was markedly smaller in hybridSPAdes than other tools, suggesting the high accuracy of its core regions were constructed from short and long reads. HybridSPAdes assembler has a higher genome fraction for each reference genome than other assemblers, whereas Flye and OPERA-MS have a higher duplication ratio. The numbers of plasmids were 15 (metaSPAdes) and 16 (hybridSPAdes) (Supplementary Table 14). The assembly statistics of the mock community supported the finding that hybrid metagenome assembly with both nanopore long- and Illumina short reads could efficiently increase assembly contiguity, and that hybridSPAdes performs better than OPERA-MS in terms of accuracy.

**FIGURE 10:**
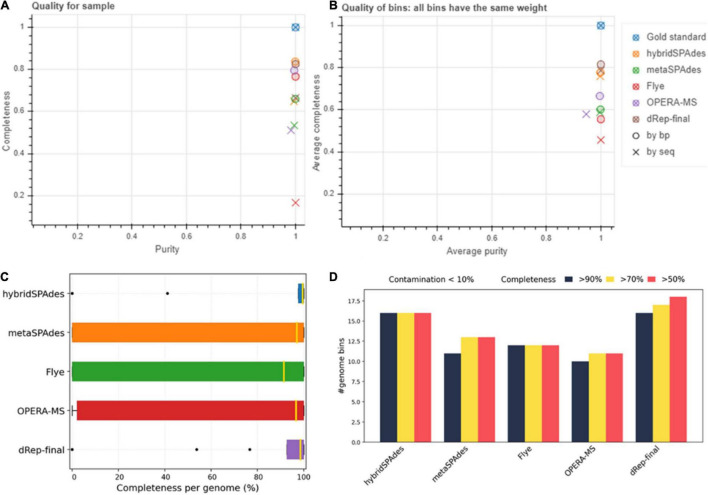
Assessment of genome bins reconstructed from mock community (BMS21) dataset by different methods. **(A)** Purity (*x*-axis) and completeness (*y*-axis). **(B)** Average purity per base pair (*x*-axis) and average completeness per base pair (*y*-axis). **(C)** Box plots of purity per bin and completeness per genome, respectively. **(D)** Number of genomes with less than 10 and 5% contamination and more than 50, 70, and 90% completeness.

Genome binning and refinement of the BMS21 mock community assembled using different software resulted in generation a total of 49 bins, including 9, 13, 16, and 11 from Flye, metaSPAdes, hybridSPAdes, and OPERA-MS assemblies, respectively (Supplementary Table 9). Binning results with the HybridSPAdes assembly algorithm were closest to the actual number of strains in the mock community, which was 21. However, dereplication (dRep) of metagenome bins from different assembly methods resulted in the generation of a total of 18 final bins (hybridSPAdes 16, metaSPAdes 4, and OPERA-MS 2). A gold standard mapping shows that MAGs generated by Flye and dRep achieved the highest purity per bin, and by dRep and hybridSPAdes achieved the highest completeness per genome on this dataset (Figures 10A,B). MAGs generated by dRep recovered the most genomes with the specified thresholds of completeness and contamination on this dataset (Figures 10C,D and Table 5). The completeness of the 18 bins ranged from 75.86 to 100%, with the majority (*n* = 16, 83%) being more than 95%. The contamination level of these bins ranged from 0 to 5.17%. The number of contigs in the 18 bins ranged from 6 to 205, with 6 (33.3%) bins containing no more than 30 contigs at more than 99.5% completeness. The abundance of the five bins was from 0.1 to 30%, which represented a majority genome content of the mock community (Supplementary Figure 5 and Supplementary Tables 10–12). ANI between metagenome bins and the individually assembled genomes ranged from 81.6 to 99.98%, with the majority (*n* = 15, 83.3%) being more than 99%. SNP of metagenome ranged from 11 to 81,540, with 7 bins (38.8%) exhibiting less than 100 SNPs compared to the reference genomes. Genome sequences of three strains in the mock community were not resolved with the algorithms, with the abundance of each genome being 0.10 and 0.25% (Supplementary Table 12), respectively. Findings here indicated the potential of hybrid genome assembly to resolve the near-complete and high-fidelity metagenome bins, and our workflow could generate more and higher quality MAGs, but there is still room for improvement of such algorithms in terms of assembly and binning accuracy.

**TABLE 5:** Respective numbers of genomes recovered from mock community (BMS21) dataset with less than 10 and 5% contamination and more than 50, 70, and 90% completeness.

Tool	Contamination	>50% completeness	>70% completeness	>90% completeness
Gold standard	<10%	21	21	21
Gold standard	<5%	21	21	21
dRep	<10%	18	17	16
dRep	<5%	18	17	16
Flye	<10%	12	12	12
Flye	<5%	12	12	12
hybridSPAdes	<10%	16	16	16
hybridSPAdes	<5%	16	16	16
metaSPAdes	<10%	13	13	11
metaSPAdes	<5%	13	13	11
OPERA-MS	<10%	11	11	10
OPERA-MS	<5%	11	11	10

## Discussion

The human gut microbiota is one of the most studied microbial environments, but technical and practical constraints hinder our ability to isolate and sequence every constituent species (Almeida et al., 2019). Currently, short-read sequencing is still one of the most cost-effective approaches to study complex microbial communities. Long-read sequencing methods (PacBio and Oxford nanopore), which have been widely applied in the study of single bacteria genomes, were gradually applied in metagenome studies (Loman et al., 2015; Jin et al., 2022). To our knowledge, although research groups have applied the third-generation long-read sequencing in metagenome-related studies (Frank et al., 2016; Tsai et al., 2016; Driscoll et al., 2017; Kerkhof et al., 2017; Bertrand et al., 2019; Overholt et al., 2020), the feasibility of nanopore sequencing in metagenomic studies remains to be unveiled, and the methods of assembling MAGs depending on a HiSeq-Nanopore hybrid metagenomic approach need to improve.

Mock communities, which represent simpler communities compared to the natural ones, are commonly recognized as a gold standard (Meyer et al., 2021) for evaluating metagenomic assemblies (Bertrand et al., 2019). By applying the workflow from metagenomic DNA analysis to generation of finally assembled bins in both natural healthy human gut microbiota and mock community, this study demonstrated the advantage of hybrid assembly with both short- and long-sequencing reads in both complex and simplified communities and the better performance of our workflow.

Specific benefits of analyzing nanopore contigs were the considerably larger average contig sizes as well as the number of large contigs, with the latter being comparable to the HiSeq assembly that was generated from tens to hundreds of folds of data. In metagenomic analyses, larger contigs are key to producing higher quality output that is needed for downstream applications such as taxonomic assignments (Patil et al., 2011; Ciuffreda et al., 2021), gene calling, annotation of operons (often exceed 10 kb in length), or detection of structural variation (Pope et al., 2010). The assembly output from both platforms varied considerably in both contig size and distribution (Figures 2, 3). Despite the similar size of the hybrid assembly and Illumina assembly contigs >0.5 kb contig datasets available for binning, the contig size of bins obtained from the Nanopore sequencing data were, on average, ∼3× to ∼6× larger, respectively (Figure 5). Another observation was the examples of hybrid contigs containing difficult to assemble regions. Hence, this approach presents an alternative means to reconstruct genomes in cases where phylotypes are not conducive to Illumina assembly alone and experimental design that cannot handle multiple sample timepoints or several differential DNA extractions, which are necessary for accurate binning algorithms that use differential coverage of populations (Alneberg et al., 2014; Imelfort et al., 2014).

This study shows the potential value that nanopore long-sequencing reads can exert upon a metagenomic study, although there is certain room for improvement. The comparative high cost of nanopore data restricts the sequencing depth of raw data used. Moreover, a major concern with the usage of nanopore reads is data wastage with respect to the number that passes the quality cutoffs. Increasing read quality and cost reductions would benefit its future applications.

We presented human gut microbiome co-assembled with Illumina short reads and nanopore long reads. Hybrid metagenome assembly resulted in a significant increase in contig length and accuracy, as well as enhancement in efficiency of taxonomic binning and genome construction compared with that using Illumina short-read data alone. OPERA-MS performs well on contig contiguity and hybridSPAdes was good at accuracy. Using our workflow, 58 high-quality metagenome bins were successfully obtained from the gut microbiota of a healthy young man, and 29 of them were currently uncultured bacteria. In summary, this study generated the high-resolution human metagenome, which could serve as a reference to improve the quality and comprehensiveness of future human metagenomics studies. Findings in this study show that nanopore long reads are highly valuable in metagenomic applications.

## Data Availability Statement

The datasets presented in this study can be found in online repositories. The names of the repository/repositories and accession number(s) can be found in the article/Supplementary Material.

## Ethics Statement

Ethical review and approval was not required for the study on human participants in accordance with the Local Legislation and Institutional Requirements. The patients/participants provided their written informed consent to participate in this study. Written informed consent was obtained from the individual(s) for the publication of any potentially identifiable images or data included in this article.

## Author Contributions

LY and ND designed and performed the experiment and data analysis, and wrote the manuscript. WX, RL, HH, and JL helped with data analysis. EC edited the manuscript. SC supervised the whole project and wrote the manuscript. All authors contributed to the article and approved the submitted version.

## Conflict of Interest

The authors declare that the research was conducted in the absence of any commercial or financial relationships that could be construed as a potential conflict of interest.

## Publisher’s Note

All claims expressed in this article are solely those of the authors and do not necessarily represent those of their affiliated organizations, or those of the publisher, the editors and the reviewers. Any product that may be evaluated in this article, or claim that may be made by its manufacturer, is not guaranteed or endorsed by the publisher.

## Footnotes

1
https://www.hmpdacc.org/catalog/

2
https://www.ebi.ac.uk/ena/browser/text-search?query=ERP108418
