# Supplementary material for: High-Resolution Metagenomics of Human Gut Microbiota Generated by Nanopore and Illumina Hybrid Metagenome Assembly
Source: Front Microbiol. 2022 May 12;13:801587. doi: 10.3389/fmicb.2022.801587 (PMC9134245; doi:10.3389/fmicb.2022.801587)
Supplement: Supplementary file 5 [file Data_Sheet_5.doc]

Supplementary materials

**High resolution metagenomics of human gut microbiota generated by Nanopore and Illumina hybrid metagenome assembly**

Lianwei YE^1#^, Ning Dong^1#^, Wenguang Xiong^2^, Jun Li^1^, Runsheng Li^1^, Heng HENG^1^, Edward Wai Chi CHAN^3^, Sheng CHEN^1*^

Running title: First high-resolution human metagenome

^1^Department of Infectious Diseases and Public Health, Jockey Club College of Veterinary Medicine and Life Sciences, City University of Hong Kong, Kowloon, Hong Kong

^2^College of Veterinary Medicine, South China Agriculture University, Guangzhou, China

^3^State Key Lab of Chemical Biology and Drug Discovery, Department of Applied Biology and Chemical Technology, The Hong Kong Polytechnic University, Hung Hom, Hong Kong;

^4^Hong Kong Branch of Southern Marine Science and Engineering Guangdong Laboratory (Guangzhou)

# Contribute equally to the work.

Corresponding author: Sheng Chen, Email: [shechen@cityu.edu.hk](mailto:shechen@cityu.edu.hk); City University of Hong Kong, Kowloon, Hong Kong

**Keywords:** Human metagenome, Illumina, Nanopore, Hybrid assembly, High resolution

**Supplementary table S1. Assembly statistics of the human gut metagenome generated with diverse softwares by metaQUAST.**

| **Genome statistics** | **hybridSPAdes** | **metaSPAdes** | **OPERA_MS** | **Flye** |
| --- | --- | --- | --- | --- |
| Genome fraction (%) | 57.726 | 55.865 | 57.448 | 42.355 |
| Duplication ratio | 1.086 | 1.033 | 1.11 | 1.324 |
| Largest aligment | 188559 | 159134 | 197506 | 363075 |
| Total aligned length | 94450111 | 90856996 | 94632968 | 56555434 |
| Misassemblies | 3092 | 2150 | 3529 | 2616 |
| Misassembled contigs length | 48249524 | 33695102 | 58256304 | 54137919 |
| mismatches per 100 kbp | 1949.44 | 1621.69 | 2310.56 | 3120.73 |
| Largest contig | 807998 | 595004 | 3008077 | 2947413 |
| N50 | 11867 | 6048 | 12770 | 227485 |
| Contigs | 131093 | 150854 | 134680 | 1968 |
| contigs (>= 0 bp) | 618388 | 652273 | 340941 | 1969 |
| contigs (>= 1000 bp) | 55677 | 66944 | 54735 | 1903 |
| contigs (>= 5000 bp) | 11151 | 11413 | 10040 | 1695 |
| contigs (>= 10000 bp) | 5505 | 5108 | 4971 | 1491 |
| contigs (>= 25000 bp) | 2088 | 1743 | 1727 | 1063 |
| contigs (>= 50000 bp) | 876 | 656 | 673 | 631 |
| NG50 |  |  |  |  |
| Anaerostipes_hadrus | 547448 | 1813 | 1084411 | 2947413 |
| Bacteroides_fragilis_str._3725_D9_ii | 360471 | 158728 | 2482251 | 2520644 |
| Bacteroides_sp._3_1_19 | 392579 | 163581 | 3008077 | 2947413 |
| Bacteroides_vulgatus | 392579 | 144329 | 3008077 | 2947413 |
| Bacteroides_vulgatus_CL09T03C04 | 354838 | 144329 | 3008077 | 2947413 |
| Butyricimonas_faecihominis | 276050 | 47228 | 3008077 | 2596188 |
| Butyricimonas_paravirosa | 321428 | 740 | 3008077 | 2339532 |
| Butyricimonas_virosa | 324899 | 4141 | 3008077 | 2947413 |
| Collinsella_aerofaciens_ATCC_25986 | 342806 | 13780 | 55305 | 2947413 |
| Escherichia_coli | 9795 | - | 457645 | 2606797 |
| Escherichia_coli_O104_H4_str._2009EL_2071 | 33100 | 11512 | 459357 | 2947413 |
| Eubacterium_rectale | 555315 | 106203 | 1092151 | 2596188 |
| Eubacterium_rectale_DSM_17629 | 514441 | 88984 | 1092151 | 2947413 |
| Faecalibacterium_prausnitzii | 578001 | 59019 | 1092151 | 2606797 |
| Flavonifractor_plautii_ATCC_29863 | 547448 | 2994 | 280774 | 2947413 |
| Fusicatenibacter_saccharivorans | 555315 | 18767 | 1092151 | 2596188 |
| Haemophilus_parainfluenzae_T3T1 | 28195 | 4534 | 562397 | 2947413 |
| Holdemanella_biformis_DSM_3989 | 699743 | 6447 | 1092151 | 2596188 |
| Klebsiella_pneumoniae | 72774 | 17245 | 459357 | 2947413 |
| Klebsiella_pneumoniae_KCTC_2242 | 57736 | 19477 | 459357 | 2947413 |
| Klebsiella_pneumoniae_subsp._pneumoniae | 44738 | 19997 | 456845 | 2947413 |
| Klebsiella_variicola | - | - | 459357 | 2947413 |
| Lactobacillus_rogosae | 281203 | 37262 | 1084411 | 2947413 |
| Megamonas_funiformis_YIT_11815 | 596353 | 65935 | 821685 | 2947413 |
| Megamonas_rupellensis | 596353 | 71499 | 821685 | 2947413 |
| Megasphaera_elsdenii | 547448 | 7045 | 50640 | 2947413 |
| Megasphaera_elsdenii_DSM_20460 | 547448 | 7045 | 50640 | 2947413 |
| Odoribacter_splanchnicus_DSM_20712 | 261287 | 99583 | 3008077 | 2339532 |
| Oscillibacter_sp._KLE_1745 | 276050 | 33843 | 229261 | 2596188 |
| Oscillospiraceae_bacterium_VE202_24 | 276050 | 100047 | 1084411 | 2596188 |
| Parabacteroides_distasonis | 339838 | 167233 | 3008077 | 2947413 |
| Parabacteroides_johnsonii_CL02T12C29 | 357220 | 169813 | 3008077 | 2947413 |
| Parabacteroides_johnsonii_DSM_18315 | 392579 | 147904 | 3008077 | 2947413 |
| Parabacteroides_merdae | 431006 | 169688 | 3008077 | 2947413 |
| Parabacteroides_merdae_CL09T00C40 | 431006 | 169688 | 3008077 | 2947413 |
| Paraprevotella_clara | 353507 | 112521 | 3008077 | 2339532 |
| Paraprevotella_xylaniphila | 392579 | 106830 | 3008077 | 2339532 |
| Phascolarctobacterium_succinatutens_YIT_12067 | 552059 | 79624 | 446400 | 2606797 |
| Romboutsia_ilealis | 354838 | - | 10074 | 2596188 |
| Roseburia_faecis | 555315 | 123066 | 1092151 | 2947413 |
| Roseburia_hominis_A2_183 | 555315 | 106253 | 1092151 | 2947413 |
| Roseburia_intestinalis_XB6B4 | 514441 | 41748 | 1144294 | 2947413 |
| Roseburia_inulinivorans | 514441 | 87252 | 1092151 | 2947413 |
| Ruminococcus_faecis | 555315 | 90603 | 1092151 | 2947413 |
| Ruminococcus_gnavus_CC55_001C | 273223 | 622 | 1084411 | 2947413 |
| Ruminococcus_lactaris_CC59_002D | 555315 | 102049 | 1092151 | 2596188 |
| Ruminococcus_sp._5_1_39BFAA | 555315 | 33116 | 1092151 | 2596188 |
| Ruminococcus_torques_L2_14 | 555315 | 64656 | 1092151 | 2947413 |
| Shigella_dysenteriae_Sd197 | 12597 | - | 546806 | 2947413 |
| Shigella_flexneri_2002017 | 17131 | - | 546806 | 2947413 |
| Streptococcus_parasanguinis_ATCC_903 | 95202 | - | - | 2947413 |
| Streptococcus_salivarius | 304009 | - | - | 2947413 |
| Streptococcus_salivarius_K12 | 304009 | - | - | 2947413 |
| Sutterella_wadsworthensis_2_1_59BFAA | 264337 | 31359 | 223209 | 2947413 |
| Veillonella_dispar_ATCC_17748 | 547448 | - | 342951 | 2947413 |
| NGA50 |  |  |  |  |
| Anaerostipes_hadrus | 947 | 741 | 574 | - |
| Bacteroides_fragilis_str._3725_D9_ii | 8941 | 11121 | 10608 | 18170 |
| Bacteroides_sp._3_1_19 | 26464 | 23989 | 26688 | 26247 |
| Bacteroides_vulgatus | 29576 | 26063 | 33233 | 34860 |
| Bacteroides_vulgatus_CL09T03C04 | 32042 | 23277 | 37078 | 36168 |
| Butyricimonas_faecihominis | 32252 | 7754 | 35562 | 2877 |
| Butyricimonas_paravirosa | - | - | - | - |
| Butyricimonas_virosa | - | - | 1197 | - |
| Collinsella_aerofaciens_ATCC_25986 | - | - | - | - |
| Escherichia_coli | - | - | 1767 | 16006 |
| Escherichia_coli_O104_H4_str._2009EL_2071 | 11556 | 9467 | 35067 | 46634 |
| Eubacterium_rectale | 19411 | 18970 | 21939 | 2028 |
| Eubacterium_rectale_DSM_17629 | 21176 | 15919 | 20878 | 4122 |
| Faecalibacterium_prausnitzii | 19315 | 8699 | 16093 | - |
| Flavonifractor_plautii_ATCC_29863 | 2310 | 1844 | 1869 | - |
| Fusicatenibacter_saccharivorans | 12309 | 6725 | 6345 | - |
| Haemophilus_parainfluenzae_T3T1 | - | - | - | - |
| Holdemanella_biformis_DSM_3989 | - | - | - | - |
| Klebsiella_pneumoniae | 17788 | 13128 | 1037 | 108559 |
| Klebsiella_pneumoniae_KCTC_2242 | 15746 | 14940 | 1140 | 103687 |
| Klebsiella_pneumoniae_subsp._pneumoniae | 19715 | 16260 | 1663 | 100642 |
| Klebsiella_variicola | - | - | 76 | 89 |
| Lactobacillus_rogosae | 18781 | 13468 | 56605 | 55228 |
| Megamonas_funiformis_YIT_11815 | 35580 | 32694 | 32856 | 36762 |
| Megamonas_rupellensis | 29376 | 15231 | 22270 | 29357 |
| Megasphaera_elsdenii | - | - | - | - |
| Megasphaera_elsdenii_DSM_20460 | - | - | - | - |
| Odoribacter_splanchnicus_DSM_20712 | 45277 | 34641 | 47614 | 20357 |
| Oscillibacter_sp._KLE_1745 | 921 | 850 | 867 | - |
| Oscillospiraceae_bacterium_VE202_24 | 1470 | 1300 | 1229 | - |
| Parabacteroides_distasonis | 24259 | 19541 | 25563 | 22990 |
| Parabacteroides_johnsonii_CL02T12C29 | - | - | - | - |
| Parabacteroides_johnsonii_DSM_18315 | - | - | - | - |
| Parabacteroides_merdae | 17018 | 12591 | 12311 | 41785 |
| Parabacteroides_merdae_CL09T00C40 | 15763 | 11628 | 11473 | 40205 |
| Paraprevotella_clara | 46683 | 39487 | 44118 | 39492 |
| Paraprevotella_xylaniphila | - | - | - | - |
| Phascolarctobacterium_succinatutens_YIT_1 | 9250 | 11862 | 8942 | 216 |
| Romboutsia_ilealis | - | - | - | - |
| Roseburia_faecis | 5261 | 4326 | 4075 | - |
| Roseburia_hominis_A2_183 | 11376 | 9694 | 8813 | - |
| Roseburia_intestinalis_XB6B4 | 4611 | 3011 | 4710 | - |
| Roseburia_inulinivorans | 14035 | 12133 | 15725 | 14213 |
| Ruminococcus_faecis | 1246 | 856 | 620 | - |
| Ruminococcus_gnavus_CC55_001C | - | - | - | - |
| Ruminococcus_lactaris_CC59_002D | - | - | - | - |
| Ruminococcus_sp._5_1_39BFAA | 10958 | 6523 | 8047 | - |
| Ruminococcus_torques_L2_14 | 1316 | 862 | 622 | - |
| Shigella_dysenteriae_Sd197 | - | - | 7821 | 15225 |
| Shigella_flexneri_2002017 | - | - | 10959 | 24085 |
| Streptococcus_parasanguinis_ATCC_903 | - | - | - | - |
| Streptococcus_salivarius | - | - | - | - |
| Streptococcus_salivarius_K12 | - | - | - | - |
| Sutterella_wadsworthensis_2_1_59BFAA | 22207 | 13650 | 21529 | 23322 |

**Supplementary table S2.** **Summary of human metagenome bins generated with diverse softwares.**

| **Total-No.bins** | **Concoct** | **Maxbin2** | **Metabat2** | **Metawrap** |
| --- | --- | --- | --- | --- |
| Flye | 1 | 0 | 0 | 0 |
| metaSPAdes | 133 | 104 | 114 | 52 |
| OPERA_MS | 1 | 89 | 110 | 43 |
| hybridSPAdes | 131 | 98 | 146 | 52 |
| Total | 266 | 291 | 370 | 147 |

**Supplementary table S3. Statistics of the plasmids (>10kb).**

| **Contig characteristics** | **metaSPAdes** | **Flye** | **OPERA-MS** | **hybridSPAdes** |
| --- | --- | --- | --- | --- |
| Sequence Number（>10k） | 65 | 129 | 164 | 174 |
| Average Length (bp) | 22399 | 26363 | 27613 | 25219 |
| The N50 Length (bp) | 25096 | 23365 | 36321 | 29233 |
| Maximum Length (bp) | 162,508 | 145,633 | 229,251 | 214,848 |

**Supplementary table S4. Size of top 10 longest plasmids generated by four programs.**

| **No.** | **Flye(bp)** | **metaSPAdes(bp)** | **OPERA-MS(bp)** | **hybridSPAdes(bp)** |
| --- | --- | --- | --- | --- |
| 1 | 162508 | 162508 | 229251 | 214848 |
| 2 | 23365 | 132508 | 211250 | 208300 |
| 3 | 20022 | 125241 | 188880 | 188603 |
| 4 | 18616 | 78942 | 162892 | 152484* |
| 5 | 15797 | 62233 | 157875* | 95856 |
| 6 | 10396 | 56159 | 137281 | 91900 |
| 7 |  | 54152 | 120319 | 70851 |
| 8 |  | 53263 | 107555 | 67793 |
| 9 |  | 49937 | 95515 | 53263 |
| 10 |  | 49820 | 95105 | 49937 |

* complete plasmids

**Supplementary table S5. Antimicrobial** resistance genes in metagenome sequences assembled with different programs.

| **Assembly programs** | **Gene ID** | **Assembly programs** | **Gene ID** |
| --- | --- | --- | --- |
|  | aac(3)-IId_1_EU022314  aadA5_1_AF137361  blaOKP-B-15_1_AM850917  blaSHV-11_4_EF035558  cfxA3_1_AF472622  dfrA17_1_FJ460238  erm(F)_3_M17808  fosA_2_AGDM01000012  fosA_7_NZ_AFBO01000747  lnu(C)_1_AY928180  oqxA_1_EU370913  oqxB_1_EU370913  strA_1_M96392  tet(40)_2_FJ158002  tet(A)_4_AJ517790  tet(C)_10_AY043299  tet(W)_6_FN396364 | hybridSPAdes（27） | sul1_1_AY224185 |
|  |  |  | sul2_2_GQ421466 |
|  |  |  | tet(32)_2_EF626943 |
|  |  |  | tet(A)_4_AJ517790 |
|  |  |  | tet(C)_10_AY043299 |
| metaSPAdes(17) |  |  | tet(O)_1_M18896 |
|  |  |  | tet(Q)_1_L33696 |
|  |  |  | tet(W)_6_FN396364 |
|  |  |  | tetA(P)_1_AB054980 |
|  |  |  | tetB(P)_3_NC_010937 |
|  |  | OPERA-MS（29） | aac(3)-IId_1_EU022314 |
|  |  |  | aac(6')-aph(2'')_1_M13771 |
|  |  |  | aac(6')-aph(2'')_1_M13771 |
|  |  |  | aadA1_1_X02340 |
|  |  |  | aadA5_1_AF137361 |
|  |  |  | blaCARB-2_1_M69058 |
|  |  |  | blaOXA-347_1_JN086160 |
|  |  |  | blaSHV-1_11_EF035565 |
|  |  |  | cat_2_M35190 |
|  |  |  | cfxA3_1_AF472622 |
|  |  |  | cfxA4_1_AY769933 |
|  |  |  | cfxA6_1_GQ342996 |
| hybridSPAdes（27） | aac(3)-IId_1_EU022314 |  | dfrA1_25_AJ844287 |
|  | aac(6')-aph(2'')_1_M13771 |  | dfrA7_1_AJ419170 |
|  | aadA5_1_AF137361 |  | erm(B)_18_X66468 |
|  | blaOKP-B-15_1_AM850917 |  | erm(F)_3_M17808 |
|  | blaSHV-11_4_EF035558 |  | fosA_9_NZ_ACZD01000244 |
|  | dfrA17_1_FJ460238 |  | lnu(C)_1_AY928180 |
|  | erm(B)_1_JN899585 |  | mph(A)_2_U36578 |
|  | erm(B)_10_U86375 |  | msr(D)_2_AF274302 |
|  | erm(F)_3_M17808 |  | oqxA_1_EU370913 |
|  | floR_2_AF118107 |  | strA_1_M96392 |
|  | fosA_2_AGDM01000012 |  | strB_1_M96392 |
|  | fosA_7_NZ_AFBO01000747 |  | sul1_2_CP002151 |
|  | lnu(C)_1_AY928180 |  | sul2_2_GQ421466 |
|  | oqxA_1_EU370913 |  | tet(A)_4_AJ517790 |
|  | oqxB_1_EU370913 |  | tet(C)_10_AY043299 |
|  | QnrS1_1_AB187515 |  | tet(Q)_3_U73497 |
|  | strA_1_M96392 |  | tetB(P)_3_NC_010937 |

**Supplementary table S6. Information of contigs carrying multiple drug resistance genes.**

| **Assembly programs** | **Contig ID** | **Size(bp)** | **Location and host of the contig** | **Genes** |
| --- | --- | --- | --- | --- |
| Flye | Flye-1 | 80893 | chromosome.Bacteroidetes | erm(F)_3_M17808,tet(Q)_3_U73497,tet(Q)_1_L33696 |
|  | Flye-2 | 3045907 | chromosome.Bacteroidetes | erm(F)_3_M17808,cfxA3_1_AF472622, |
| metaSPAdes | metaSPAdes-1 | 62014 | chromosome.Proteobacteria | oqxA_1_EU370913,oqxB_1_EU370913, |
|  | metaSPAdes-2 | 1615 | plasmid.Proteobacteria | dfrA17_1_FJ460238,aadA5_1_AF137361, |
| hybridSPAdes | hybridSPAdes-1 | 61575 | chromosome.Proteobacteria | oqxB_1_EU370913,oqxA_1_EU370913, |
|  | hybridSPAdes-2 | 27854 | plasmid.Proteobacteria | tet(A)_4_AJ517790,strB_1_M96392,strA_1_M96392,sul2_2_GQ421466 |
|  | hybridSPAdes-3 | 1615 | plasmid.Proteobacteria | dfrA17_1_FJ460238,aadA5_1_AF137361, |
|  | hybridSPAdes-4 | 12640 | plasmid.Firmicutes | erm(B)_18_X66468,aac(6')-aph(2'')_1_M13771, |
|  | hybridSPAdes-5 | 9417 | plasmid.Firmicutes | tetB(P)_3_NC_010937,tetA(P)_1_AB054980, |
| OPERA-MS | OPERA-MS-1 | 17410 | plasmid.Proteobacteria | tet(R)_6_Y19114,lnu(C)_1_AY928180,sul1_2_CP002151,aadA1_1_X02340,tet(A) |
|  | OPERA-MS-2 | 15762 | plasmid.Proteobacteria | tet(A)_4_AJ517790,strB_1_M96392,strA_1_M96392 |
|  | OPERA-MS-3 | 1703 | plasmid.Proteobacteria | aadA5_1_AF137361,dfrA7_1_AJ419170, |
|  | OPERA-MS-4 | 67611 | plasmid.Proteobacteria | dfrA1_25_AJ844287,blaCARB-2_1_M69058, |

**Supplementary table S7. Genome statistics of contigs for the mock community generated with diverse software by metaQUAST.**

| **Genome statistics** | **hybridSPAdes** | **metaSPAdes** | **Flye** | **OPERA_MS** |
| --- | --- | --- | --- | --- |
| 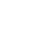 contigs | 1812 | 2999 | 229 | 2521 |
| # contigs (>= 1000 bp) | 1255 | 2302 | 226 | 1703 |
| # contigs (>= 5000 bp) | 874 | 1516 | 204 | 876 |
| # contigs (>= 10000 bp) | 720 | 1171 | 186 | 587 |
| # contigs (>= 25000 bp) | 509 | 716 | 154 | 303 |
| # contigs (>= 50000 bp) | 325 | 437 | 106 | 169 |
| N50 | 209,776 | 93,165 | 4,185,707 | 385,369 |
| max_len | 2,247,228 | 670,411 | 6,834,171 | 6,176,973 |
| sum_len | 95,667,833 | 94,817,799 | 73,164,634 | 92,785,143 |

**Supplementary table S8. Genome statistics of assembled-contig by diverse softwares for the mock community.**

| **Assembled-Method** | **Seq #** | **Seq len** | **Seq N50** | **Seq N90** | **Ctg #** | **Ctg len** | **Ctg N50** | **Ctg N90** |
| --- | --- | --- | --- | --- | --- | --- | --- | --- |
| Flye | 47 | 19695577 | 2456013 | 139451 | 47 | 19695577 | 2456013 | 139451 |
| metaSPAdes | 3063 | 82931661 | 108570 | 15134 | 3479 | 82911463 | 93165 | 13043 |
| hybridSPAdes | 1607 | 85743097 | 718128 | 36395 | 1693 | 85360430 | 624915 | 34176 |
| OPERA_MS | 6528 | 63939879 | 115123 | 3788 | 13561 | 63932846 | 18108 | 1556 |

Seq #: Scaffold Number; Seq len: Scaffold Length; Seq N90: the N90 Length; Seq N50: the N50 Length; Ctg #: contig Number; Ctg len: contig Length; Ctg N90: the contig N90 Length; Ctg N50: the contig N50 Length

**Supplementary table S9.** **Summary of the mock community bins generated with diverse softwares.**

| **Total-No.bins** | **Concoct** | **Maxbin2** | **Metabat2** | **Metawrap** |
| --- | --- | --- | --- | --- |
| Flye | 27 | 13 | 28 | 9 |
| metaSPAdes | 37 | 20 | 47 | 13 |
| OPERA_MS | 52 | 15 | 23 | 11 |
| hybridSPAdes | 35 | 15 | 63 | 16 |
| Total | 151 | 63 | 161 | 49 |

**Supplementary table S10. Genome statistics of bins and genomes from the mock community.**

| **Source** | **Sample** | **num_seqs** | **sum_len** | **min_len** | **avg_len** | **max_len** | **N50** |
| --- | --- | --- | --- | --- | --- | --- | --- |
| individual assembly | 101-sz-unicycler.fasta | 2 | 4,961,339 | 111,006 | ######## | 4,850,333 | 4,850,333 |
| individual assembly | 110-sz-unicycler.fasta | 2 | 4,625,103 | 90,087 | ######## | 4,535,016 | 4,535,016 |
| individual assembly | 114-SZ-unicycler.fasta | 1 | 5,262,505 | 5,262,505 | 5,262,505 | 5,262,505 | 5,262,505 |
| individual assembly | 118-sz-unicycler.fasta | 3 | 4,511,553 | 2,683 | 1,503,851 | 4,293,241 | 4,293,241 |
| individual assembly | 158-SZ-unicycler.fasta | 2 | 4,262,912 | 2,683 | 2,131,456 | 4,260,229 | 4,260,229 |
| individual assembly | 1-E-unicycler.fasta | 20 | 5,268,789 | 126 | ######## | 4,438,248 | 4,438,248 |
| individual assembly | 20-h-unicycler.fasta | 4 | ####### | 3,146 | ####### | ####### | 2,265,484 |
| individual assembly | 274-sz-unicycler.fasta | 3 | ####### | 2,683 | ####### | ####### | 4,196,724 |
| individual assembly | 70-kc-unicycler.fasta | 5 | ####### | 6,365 | ####### | ####### | 4,654,384 |
| individual assembly | A102-unicycler.fasta | 3 | ####### | 53,827 | ####### | ####### | 3,189,772 |
| individual assembly | A103-unicycler.fasta | 2 | 2,809,270 | 46,979 | 1,404,635 | 2,762,291 | 2,762,291 |
| individual assembly | A104-unicycler.fasta | 4 | 3,169,612 | 38,288 | 792,403 | 3,014,099 | 3,014,099 |
| individual assembly | A105-unicycler.fasta | 3 | 3,049,036 | 41,596 | ######## | 2,940,724 | 2,940,724 |
| individual assembly | A106-unicycler.fasta | 5 | 5,547,682 | 13,285 | ######## | 5,303,032 | 5,303,032 |
| individual assembly | A107-unicycler.fasta | 1 | 4,870,259 | 4,870,259 | 4,870,259 | 4,870,259 | 4,870,259 |
| individual assembly | A108-unicycler.fasta | 5 | 5,209,638 | 1,919 | ######## | 5,132,005 | 5,132,005 |
| individual assembly | A110-unicycler.fasta | 1 | 6,839,764 | 6,839,764 | 6,839,764 | 6,839,764 | 6,839,764 |
| individual assembly | A111-unicycler.fasta | 1 | 6,159,443 | 6,159,443 | 6,159,443 | 6,159,443 | 6,159,443 |
| individual assembly | A112-unicycler.fasta | 3 | 3,998,073 | 7,655 | 1,332,691 | 3,980,878 | 3,980,878 |
| individual assembly | R4-unicycler.fasta | 10 | 4,694,909 | 156 | ######## | 3,045,793 | 3,045,793 |
| individual assembly | R5-unicycler.fasta | 2 | 3,044,054 | 66,795 | 1,522,027 | 2,977,259 | 2,977,259 |
| MAGs | hybridSPAdes10.fa | 32 | 4,694,530 | 2,956 | ######## | 617,480 | 303,782 |
| MAGs | hybridSPAdes12.fa | 12 | 6,795,042 | 25,754 | ######## | 2,247,228 | 1,446,274 |
| MAGs | hybridSPAdes13.fa | 33 | 4,211,854 | 2,301 | ######## | 841,606 | 312,001 |
| MAGs | hybridSPAdes14.fa | 47 | 4,797,361 | 2,690 | ######## | 498,031 | 201,414 |
| MAGs | hybridSPAdes15.fa | 12 | 5,245,521 | 27,692 | ######## | 1,651,606 | 700,786 |
| MAGs | hybridSPAdes16.fa | 14 | 4,225,140 | 15,790 | ######## | 1,129,770 | 558,433 |
| MAGs | hybridSPAdes1.fa | 91 | 3,033,236 | 1,426 | 33,332.30 | 175,186 | 52,217 |
| MAGs | hybridSPAdes2.fa | 24 | 4,582,133 | 34,153 | ######## | 466,949 | 267,896 |
| MAGs | hybridSPAdes3.fa | 13 | 6,139,906 | 31,424 | ######## | 2,060,591 | 1,463,729 |
| MAGs | hybridSPAdes4.fa | 50 | 3,004,108 | 1,101 | 60,082.20 | 396,204 | 143,958 |
| MAGs | hybridSPAdes5.fa | 37 | 4,975,892 | 2,334 | ######## | 517,879 | 251,374 |
| MAGs | hybridSPAdes7.fa | 7 | 2,732,822 | 38,599 | ######## | 861,752 | 835,157 |
| MAGs | metaSPAdes.10.fa | 205 | 2,958,743 | 1,035 | 14,432.90 | 103,136 | 23,209 |
| MAGs | metaSPAdes.11.fa | 103 | 4,810,978 | 1,025 | 46,708.50 | 390,163 | 180,136 |
| MAGs | metaSPAdes.3.fa | 27 | 2,146,719 | 3,590 | 79,508.10 | 382,459 | 191,740 |
| MAGs | metaSPAdes.5.fa | 54 | 2,802,948 | 4,151 | 51,906.40 | 382,859 | 86,547 |
| MAGs | opera-hybrid1.fa | 6 | 4,007,120 | 6,822 | ######## | 1,423,092 | 1,304,295 |
| MAGs | opera-hybrid2.fa | 51 | 4,348,352 | 2,952 | 85,261.80 | 313,168 | 161,041 |
| MAGs | opera-hybrid5.fa | 13 | 2,944,496 | 5,488 | ######## | 914,757 | 400,895 |

**Supplementary table S11. The ANI and SNP statistics of bins and genomes for the mock community.**

| Sequencing- ID | species | ID | source | mixed% | assembled-bin ID | ANI% | SNP NO. |
| --- | --- | --- | --- | --- | --- | --- | --- |
| 101-sz | *Enterobacter asburiae* | Ea101 | pig sample | 1.50% | hybridSPAdes-metabat2-bin.22.contigs | 99.09% | 3573 |
| 110-sz | *Hafnia alvei* | Ha110 | pig sample | 3.00% | hybridSPAdes-concoct-bin.16 | 99.99% | 55 |
| 114-sz | *Serratia liquefaciens* | Sl114 | pig sample | 20.00% | hybridSPAdes-maxbin2-bin.3_sub.contigs | 99.99% | 3 |
| 118-sz | *Providencia rettgeri* | Pr118 | pig sample | 0.25% | hybridSPAdes-concoct-bin.35_sub.contigs | 99.97% | 564 |
| 158-sz | *Providencia heimbachae* | Ph158 | pig sample | 15.00% | hybridSPAdes-concoct-bin.15.contigs | 99.99% | 1475 |
| 1-E | *Escherichia.coli* | Ec1 | human | 0.25% | hybridSPAdes-concoct-bin.39.contigs | 98.98% | 29461 |
| 20-h | *Ideonella dechloratans* | Id20 | human | 1.00% | hybridSPAdes-maxbin2-bin.11_sub.contigs | 99.98% | 39 |
| 274-sz | *Morqanella morqanii* | Mm274 | pig sample | 7.00% | hybridSPAdes-concoct-bin.28 | 99.99% | 20 |
| 70-kc | *Escherichia.cloacae* | Ec70 | CRE | 0.10% | hybridSPAdes-maxbin2-bin.12_sub | 87.02% | 82747 |
| A102 | *Vibrio vulnificus* | VV102 | shrimp sample | 0.10% | hybridSPAdes-metabat2-bin.17.contigs | 99.96% | 994 |
| A103 | *Staphylococcus aureus* | 29213 | ATCC | 0.10% | hybridSPAdes-maxbin2-bin.19_sub.contigs | 99.99% | 10 |
| A104 | *Streptococcus Faecalis* | Sf104 | unknown | 0.25% | None |  |  |
| A105 | *Enterococcus faecium* | 29212 | ATCC | 0.50% | hybridSPAdes-concoct-bin.37.contigs | 99.97% | 289 |
| A106 | *Klebsiella pneumoniae* | 13883 | ATCC | 0.10% | None |  |  |
| A107 | *Salmonella typhimurium* | PY01 | unknown | 0.50% | None |  |  |
| A108 | *Escherichia coli* | 25922 | ATCC | 3.00% | None |  |  |
| A110 | *Pseudomonas aeruginosa* | 27853 | ATCC | 30.00% | hybridSPAdes-concoct-bin.27.contigs | 99.99% | 2 |
| A111 | *Pseudomonas putida* | 12633 | ATCC | 5.00% | hybridSPAdes-maxbin2-bin.6.contigs | 99.99% | 23 |
| A112 | *Acinetobacter baumannii* | 19606 | ATCC | 1.00% | hybridSPAdes-maxbin2-bin.4_sub.contigs | 99.82% | 0 |
| R4 | *Lactobacillus casei* | 393 | ATCC | 1.50% | hybridSPAdes-maxbin2-bin.15.contigs | 97.34% | 7 |
| R5 | *Lactobacillus spp* | LsR5 | yogurt | 10.00% | hybridSPAdes-concoct-bin.40.contigs | 99.99% | 19 |

**Supplementary table S12. General genomic features of mock community bins reconstructed from dereplication of metagenome assembly with different algorithms.**

| **ID** | **species** | **ID** | **source** | **mixed%** | **assembled-bin ID** | **ANI%** | **SNP NO.** | **Completeness** | **Contamination** | **avg_len** | **max_len** | **N50** |
| --- | --- | --- | --- | --- | --- | --- | --- | --- | --- | --- | --- | --- |
| 101-sz | *Enterobacter asburiae* | Ea101 | pig sample | 1.50% | metaSPAdes.11.fa | 99.9152 | 847 | 97.72 | 0.35 | 46,708.50 | 390,163 | 180,136 |
| 110-sz | *Hafnia alvei* | Ha110 | pig sample | 3.00% | hybridSPAdes2.fa | 99.9486 | 377 | 99.62 | 0.45 | 190,922.20 | 466,949 | 267,896 |
| 114-sz | *Serratia liquefaciens* | Sl114 | pig sample | 20.00% | hybridSPAdes15.fa | 99.985 | 73 | 100 | 0.45 | 437,126.80 | 1,651,606 | 700,786 |
| 118-sz | *Providencia rettgeri* | Pr118 | pig sample | 0.25% | opera-hybrid2.fa | 99.8818 | 312 | 94.26 | 1.08 | 85,261.80 | 313,168 | 161,041 |
| 158-sz | *Providencia heimbachae* | Ph158 | pig sample | 15.00% | hybridSPAdes13.fa | 99.9773 | 55 | 99.22 | 0 | 127,631.90 | 841,606 | 312,001 |
| 1-E | *Escherichia.coli* | Ec1 | human | 0.25% | hybridSPAdes10.fa | 98.7691 | 25385 | 97.06 | 0.22 | 146,704.10 | 617,480 | 303,782 |
| 20-h | *Ideonella dechloratans* | Id20 | human | 1.00% | metaSPAdes.3.fa | 99.9702 | 11 | 98.47 | 0.61 | 79,508.10 | 382,459 | 191,740 |
| 274-sz | *Morqanella morqanii* | Mm274 | pig sample | 7.00% | hybridSPAdes16.fa | 99.9763 | 129 | 98.67 | 0 | 301,795.70 | 1,129,770 | 558,433 |
| 70-kc | *Escherichia.cloacae* | Ec70 | CRE | 0.10% | metaSPAdes.11.fa | 88.4221 | 81540 | 97.72 | 0.35 | 46,708.50 | 390,163 | 180,136 |
| A102 | *Vibrio vulnificus* | VV102 | shrimp sample | 0.10% | hybridSPAdes5.fa | 99.9778 | 22 | 99.73 | 0 | 134,483.60 | 517,879 | 251,374 |
| A103 | *Staphylococcus aureus* | 29213 | ATCC | 0.10% | hybridSPAdes7.fa | 99.9827 | 36 | 99.51 | 0.08 | 390,403.10 | 861,752 | 835,157 |
| A104 | *Streptococcus Faecalis* | Sf104 | unknown | 0.25% | metaSPAdes.10.fa | 99.8435 | 15946 | 98.88 | 0.37 | 14,432.90 | 103,136 | 23,209 |
| A105 | *Enterococcus faecium* | 29212 | ATCC | 0.50% | hybridSPAdes1.fa | 99.9601 | 131 | 99.63 | 0.75 | 33,332.30 | 175,186 | 52,217 |
| A106 | *Klebsiella pneumoniae* | 13883 | ATCC | 0.10% | hybridSPAdes10.fa | 81.5946 | 8290 | 97.06 | 0.22 | 146,704.10 | 617,480 | 303,782 |
| A107 | *Salmonella typhimurium* | PY01 | ATCC | 0.50% | hybridSPAdes14.fa | 99.8318 | 2109 | 75.86 | 5.17 | 102,071.50 | 498,031 | 201,414 |
| A108 | *Escherichia coli* | 25922 | ATCC | 3.00% | hybridSPAdes10.fa | 99.9471 | 482 | 97.06 | 0.22 | 146,704.10 | 617,480 | 303,782 |
| A110 | *Pseudomonas aeruginosa* | 27853 | ATCC | 30.00% | hybridSPAdes12.fa | 99.985 | 22 | 99.66 | 0.11 | 566,253.50 | 2,247,228 | 1,446,274 |
| A111 | *Pseudomonas putida* | 12633 | ATCC | 5.00% | hybridSPAdes3.fa | 99.9594 | 388 | 99.88 | 1.14 | 472,300.50 | 2,060,591 | 1,463,729 |
| A112 | *Acinetobacter baumannii* | 19606 | ATCC | 1.00% | opera-hybrid1.fa | 99.9366 | 1024 | 99.63 | 0 | 667,853.30 | 1,423,092 | 1,304,295 |
| R4 | *Lactobacillus casei* | 393 | ATCC | 1.50% | metaSPAdes.5.fa | 98.4237 | 15871 | 98.91 | 0 | 51,906.40 | 382,859 | 86,547 |
| R5 | *Lactobacillus spp* | LsR5 | yogurt | 10.00% | hybridSPAdes4.fa | 99.9827 | 78 | 98.37 | 0 | 60082.2 | 396204 | 143958 |
| R5 | *Lactobacillus spp* | LsR5 | yogurt | 0.1 | metaSPAdes.5.fa | 99.93 | 13 | 98.91 | 0 | 51906.4 | 382859 | 86547 |

**Supplementary table S13. Summary of server and running time of assemblers**

| Data type | Size | Assemblier | Hours | CPU | RAM |
| --- | --- | --- | --- | --- | --- |
| NGs | 26Gb fastq | metaSPAdes | 20 | Intel(R) Xeon(R) Platinum 8168 CPU @ 2.70GHz | 83Gb |
| NGs+nanopore | 26Gb fastq+5.4Gb fasta | metaSPAdes | 30 |  | 54Gb |
| NGs+nanopore | 26Gb fastq+5.4Gb fasta | OPERA-ms | 29 |  | 20Gb |
| Nanopore | 5.4Gb fasta | Flye | 10 |  | 35Gb |

NGs: Illumina data

**Supplementary table S14 Plasmids statistics of the mock community**

| methods | contigs | Plasmid |
| --- | --- | --- |
| metaSPAdes | NODE_1007_length_13966_cov_10.948099 | pSM22_1__NC_015972 |
|  | NODE_1025_length_13340_cov_271.242454 | ColRNAI_1__DQ298019 |
|  | NODE_1181_length_9724_cov_14.830075 | IncFIB(AP001918)_1__AP001918 |
|  | NODE_1188_length_9541_cov_21.242779 | IncFIB(K)_1_Kpn3_JN233704 |
|  | NODE_1280_length_7918_cov_21.691721 | IncFII_1__AY458016 |
|  | NODE_1738_length_3227_cov_6773.083228 | ColRNAI_1__DQ298019 |
|  | NODE_1811_length_2738_cov_57762.143496 | Col3M_1__JX514065 |
|  | NODE_237_length_92393_cov_30.951602 | IncFII_1__AY458016 |
|  | NODE_362_length_60241_cov_45.837504 | IncFIB(pHCM2)_1_pHCM2_AL513384 |
|  | NODE_378_length_57932_cov_163.264060 | IncFII(Y)_1_ps_CP001049 |
|  | NODE_610_length_32267_cov_56.911182 | IncX3_1__JN247852 |
|  | NODE_690_length_26537_cov_587.206102 | IncX1_4__JN935898 |
|  | NODE_734_length_23949_cov_24.197707 | IncFIB(pECLA)_1_pECLA_CP001919 |
|  | NODE_777_length_21775_cov_24.074954 | IncFII(pECLA)_1_pECLA_CP001919 |
|  | NODE_784_length_21357_cov_23.100084 | IncFIA(HI1)_1_HI1_AF250878 |
| HybridSPAdes | NODE_1010_length_2738_cov_57753.198286 | Col3M_1__JX514065 |
|  | NODE_191_length_111072_cov_171.834485 | IncFII(Y)_1_ps_CP001049 |
|  | NODE_219_length_91848_cov_33.236500 | IncFIB(S)_1__FN432031 |
|  | NODE_282_length_60241_cov_45.837454 | IncFIB(pHCM2)_1_pHCM2_AL513384 |
|  | NODE_317_length_50925_cov_96.386121 | IncX3_1__JN247852 |
|  | NODE_350_length_44826_cov_22.559536 | IncFIA(HI1)_1_HI1_AF250878 |
|  | NODE_504_length_25443_cov_13.531905 | pSM22_1__NC_015972 |
|  | NODE_522_length_23947_cov_24.059392 | IncFIB(pECLA)_1_pECLA_CP001919 |
|  | NODE_538_length_21933_cov_581.866898 | IncX1_4__JN935898 |
|  | NODE_541_length_21775_cov_24.074954 | IncFIB(pENTE01)_1_pENTE01_CP000654 |
|  | NODE_584_length_18676_cov_104.745395 | IncFIB(AP001918)_1__AP001918 |
|  | NODE_653_length_13796_cov_20.379594 | IncFIB(K)_1_Kpn3_JN233704 |
|  | NODE_655_length_13758_cov_19.538130 | IncFII_1__AY458016 |
|  | NODE_661_length_13340_cov_271.242303 | ColRNAI_1__DQ298019 |
|  | NODE_735_length_9281_cov_14.108173 | IncFII_1__AY458016 |
|  | NODE_973_length_3227_cov_6774.607503 | ColRNAI_1__DQ298019 |
|  |  |  |

**Supplementary table S15 Genome statistics of assembled-contig by diverse softwares for the mock community.**

| ID | **Genome fraction (%)** | | | | |  | **Duplication ratio** | | | |  | **NGA50** | | | |  | **misassemblies** | | | |
| --- | --- | --- | --- | --- | --- | --- | --- | --- | --- | --- | --- | --- | --- | --- | --- | --- | --- | --- | --- | --- |
|  | MK-h | MK-m | Mk-F | Mk-O | |  | MK-h | MK-m | Mk-F | Mk-O |  | MK-h | MK-m | Mk-F | Mk-O |  | MK-h | MK-m | Mk-F | Mk-O |
| 110-sz-unicycler | 99.74 | 99.37 | 100.00 | | 98.70 |  | 1.02 | 1.01 | 1.03 | 2.40 |  | 267409 | 201606 | 4527412 | 4397264 |  | 0 | 1 | 0 | 0 |
| 114-SZ-unicycler | 99.86 | 99.16 | 100.00 | | 31.86 |  | 1.02 | 1.01 | 1.03 | 4.81 |  | 700786 | 291582 | 5253477 | - |  | 0 | 0 | 0 | 7 |
| 118-sz-unicycler | 99.12 | 96.64 | 85.38 | | 96.99 |  | 1.02 | 1.01 | 1.05 | 2.46 |  | 141289 | 33462 | 175508 | 146522 |  | 0 | 0 | 3 | 1 |
| 158-SZ-unicycler | 99.64 | 98.86 | 99.93 | | 0.97 |  | 1.02 | 1.04 | 1.07 | 158.47 |  | 312001 | 185920 | 4247522 | - |  | 0 | 0 | 0 | 0 |
| 1-E-unicycler | 97.99 | 96.93 | 94.51 | | 91.44 |  | 1.07 | 1.04 | 1.08 | 2.49 |  | 96752 | 56878 | 130836 | 222457 |  | 91 | 51 | 104 | 58 |
| 20-h-unicycler | 99.60 | 96.78 | 99.86 | | 96.45 |  | 1.01 | 1.00 | 1.02 | 1.29 |  | 346906 | 144879 | 2262522 | 1283406 |  | 0 | 0 | 2 | 1 |
| 274-sz-unicycler | 99.63 | 99.03 | 99.94 | | 90.51 |  | 1.02 | 1.00 | 1.03 | 2.62 |  | 553831 | 356486 | 4185703 | 2571554 |  | 0 | 0 | 0 | 1 |
| 70-kc-unicycler | 87.46 | 84.06 | 34.92 | | 97.27 |  | 1.06 | 1.02 | 1.21 | 2.46 |  | 38520 | 25304 | - | 33905 |  | 1 | 0 | 3 | 2 |
| A102-unicycler | 98.44 | 97.58 | 15.84 | | 96.31 |  | 1.00 | 1.00 | 1.05 | 2.09 |  | 251374 | 90824 | - | 29854 |  | 0 | 0 | 0 | 6 |
| A103-unicycler | 99.07 | 98.58 | 83.25 | | 98.54 |  | 1.00 | 1.00 | 1.01 | 1.05 |  | 835157 | 260972 | 118296 | 502575 |  | 0 | 0 | 4 | 1 |
| A104-unicycler | 97.51 | 96.99 | 97.08 | | 93.53 |  | 1.02 | 1.01 | 1.06 | 1.13 |  | 30537 | 20864 | 70942 | 176641 |  | 33 | 4 | 56 | 32 |
| A105-unicycler | 98.68 | 98.11 | 99.55 | | 94.54 |  | 1.02 | 1.02 | 1.06 | 1.14 |  | 48328 | 22580 | 657779 | 173665 |  | 8 | 5 | 21 | 26 |
| A106-unicycler | 97.82 | 96.71 | 11.18 | | 97.18 |  | 1.04 | 1.02 | 1.41 | 2.27 |  | 44826 | 37701 | - | 20874 |  | 3 | 5 | 4 | 5 |
| A107-unicycler | 98.79 | 97.42 | 99.86 | | 97.49 |  | 1.04 | 1.01 | 1.04 | 2.42 |  | 193261 | 77015 | 2480577 | 694463 |  | 0 | 0 | 1 | 6 |
| A108-unicycler | 99.69 | 98.33 | 99.89 | | 91.74 |  | 1.06 | 1.03 | 1.11 | 2.50 |  | 303782 | 99977 | 1987073 | 3157262 |  | 1 | 2 | 8 | 9 |
| A110-unicycler | 99.50 | 98.70 | 100.00 | | 0.07 |  | 1.01 | 1.00 | 1.01 | 1201.91 |  | 1446274 | 237789 | 6834171 | - |  | 1 | 0 | 0 | 0 |
| A111-unicycler | 99.77 | 98.48 | 100.00 | | 95.86 |  | 1.00 | 1.00 | 1.01 | 1.38 |  | 1462955 | 203131 | 6152575 | 5765835 |  | 0 | 0 | 0 | 0 |
| A112-unicycler | 98.76 | 98.48 | 99.01 | | 98.31 |  | 1.00 | 1.00 | 1.01 | 1.06 |  | 385059 | 217726 | 3932066 | 1223388 |  | 1 | 1 | 2 | 4 |
| R4-unicycler | 95.52 | 91.91 | 97.44 | | 43.24 |  | 1.04 | 1.01 | 1.08 | 2.35 |  | 41908 | 24320 | 73863 | - |  | 141 | 95 | 194 | 37 |
| R5-unicycler | 98.98 | 97.01 | 100.00 | | 10.86 |  | 1.05 | 1.01 | 1.13 | 9.06 |  | 143958 | 81698 | 2292142 | - |  | 10 | 0 | 106 | 21 |
| 101-sz-unicycler | 99.23 | 98.64 | 99.91 | | 97.47 |  | 1.22 | 1.17 | 1.08 | 2.43 |  | 177736 | 135322 | 1532640 | 193371 |  | 9 | 8 | 8 | 2 |

MK-h:mock community assembled by hybridSPAdes; MK-m:mock community assembled by metaSPAdes; Mk-F:mock community assembled by Flye; Mk-O:mock community assembled by OPERA-MS

**Supplementary table S16 Respective number of genomes recovered from BMS21 dataset with less than 10% and 5% contamination and more than 50%, 70%, and 90% completeness.**

| Tool | Contamination | > 50% completeness | > 70% completeness | > 90% completeness |
| --- | --- | --- | --- | --- |
| Gold standard | < 10% | 21 | 21 | 21 |
| Gold standard | < 5% | 21 | 21 | 21 |
| dRep | < 10% | 18 | 17 | 16 |
| dRep | < 5% | 18 | 17 | 16 |
| Flye | < 10% | 12 | 12 | 12 |
| Flye | < 5% | 12 | 12 | 12 |
| hybridSPAdes | < 10% | 16 | 16 | 16 |
| hybridSPAdes | < 5% | 16 | 16 | 16 |
| metaSPAdes | < 10% | 13 | 13 | 11 |
| metaSPAdes | < 5% | 13 | 13 | 11 |
| OPERA-MS | < 10% | 11 | 11 | 10 |
| OPERA-MS | < 5% | 11 | 11 | 10 |

**
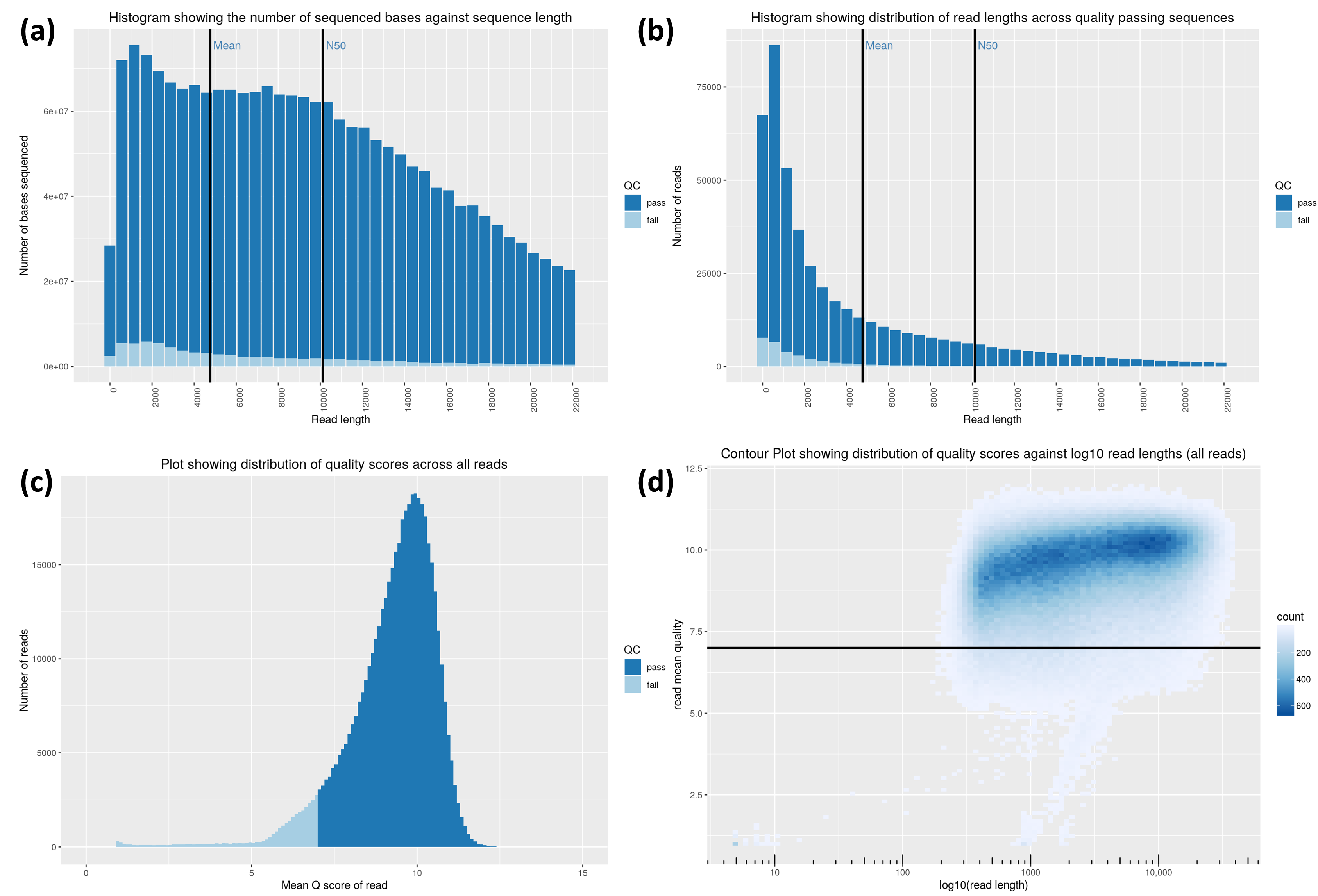
**

**Supplementary Figure S1. Statistics of an 8-hour MinION nanopore sequencing run using the Rapid Barcoding Sequencing Kit**. (A) Distribution of read length and data volume generated by the MinION run in 8 hours. (B) Total base length and read number of the 12 samples after de-multiplexing. (C) Distribution of mean read quality scores across the whole sequence collection. The distribution has been shaded for the sequence reads that have passed or failed the base-callers quality filter. (D) The density plot of mean sequence quality plotted against log10 sequence length is a useful graphic to show patterns within the broader sequence collection. The density plot shown in the figure below has been de-speckled by omitting the rarer sequence bins containing only 5 reads or fewer. This is mainly aesthetic and masks some speckle around the periphery of the main density map.

**
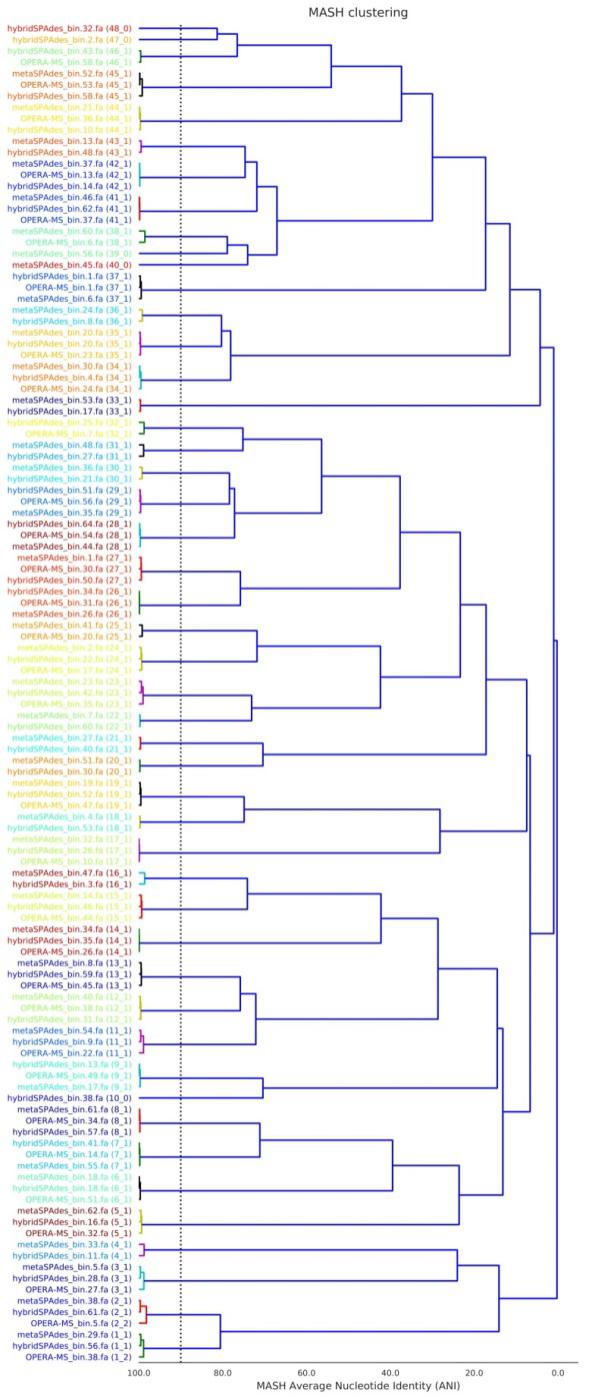
**

**Supplementary Figure S2. Dereplication of the human gut microbime bins.** The primary clustering dendrogram summarizes the pair-wise Mash distance between all genomes in the genome list. The dotted line provides a visualization of the primary ANI - the value which determines the creation of primary clusters. It is drawn in the above figure at 90% ANI (the default value).


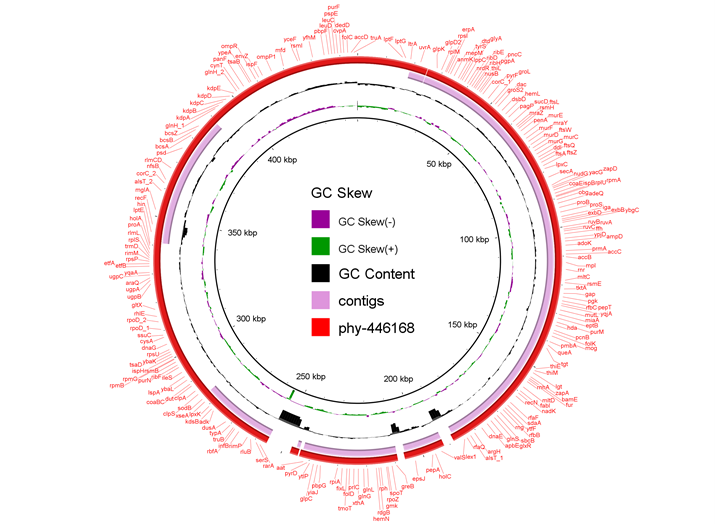


**Supplementary Figure S3. Alignment of the largest plasmids sequence by hybridSPAdes, Plasmids phy-214848, with contigs from metaSPAdes assembler.**

**
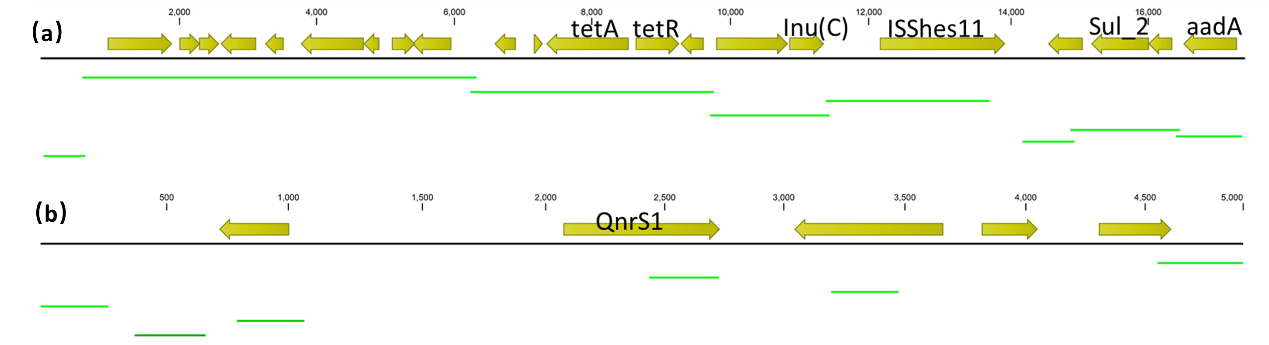
**

**Supplementary Figure S4. Comparison of gene cassettes carrying MDR genes assembled by different assembly methods.** (A) BLASTN of a 17,410 bp contig carrying multiple drug resistance genes assembled using OPERA-MS against that of metaSPAdes assembly. The result shows that hybrid assembly with OPERA-MS generate contiguous contig carring MDR genes compared to assembly with illumina reads alone (B) BLASTN of a 4,910 bp contig hybrid assembled using hybridSPAdes against the metaSPAdes assembly with illumina reads alone. The *qnrS1* gene was only identified in hybridSPAdes contigs. This result shows the resistance gene could be assembled by hybridSPAdes assembler.

**
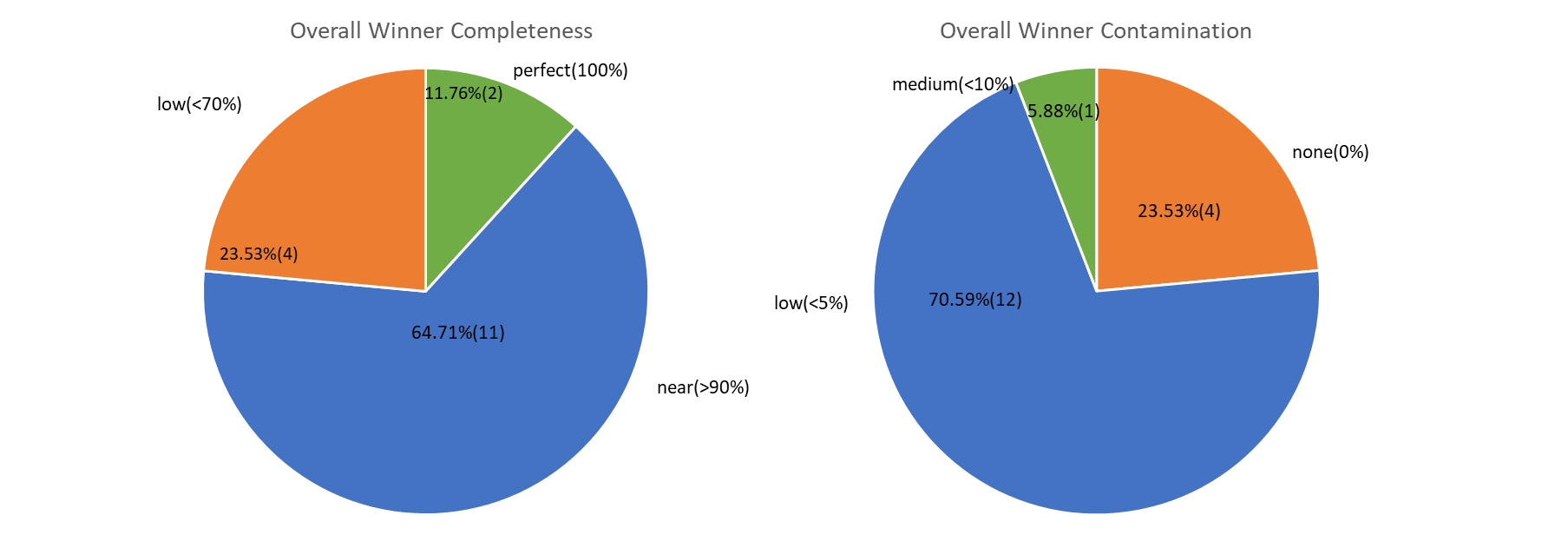
**

**Supplementary Figure S5. Distribution of completeness and contamination of the mock community bins after dereplication.**


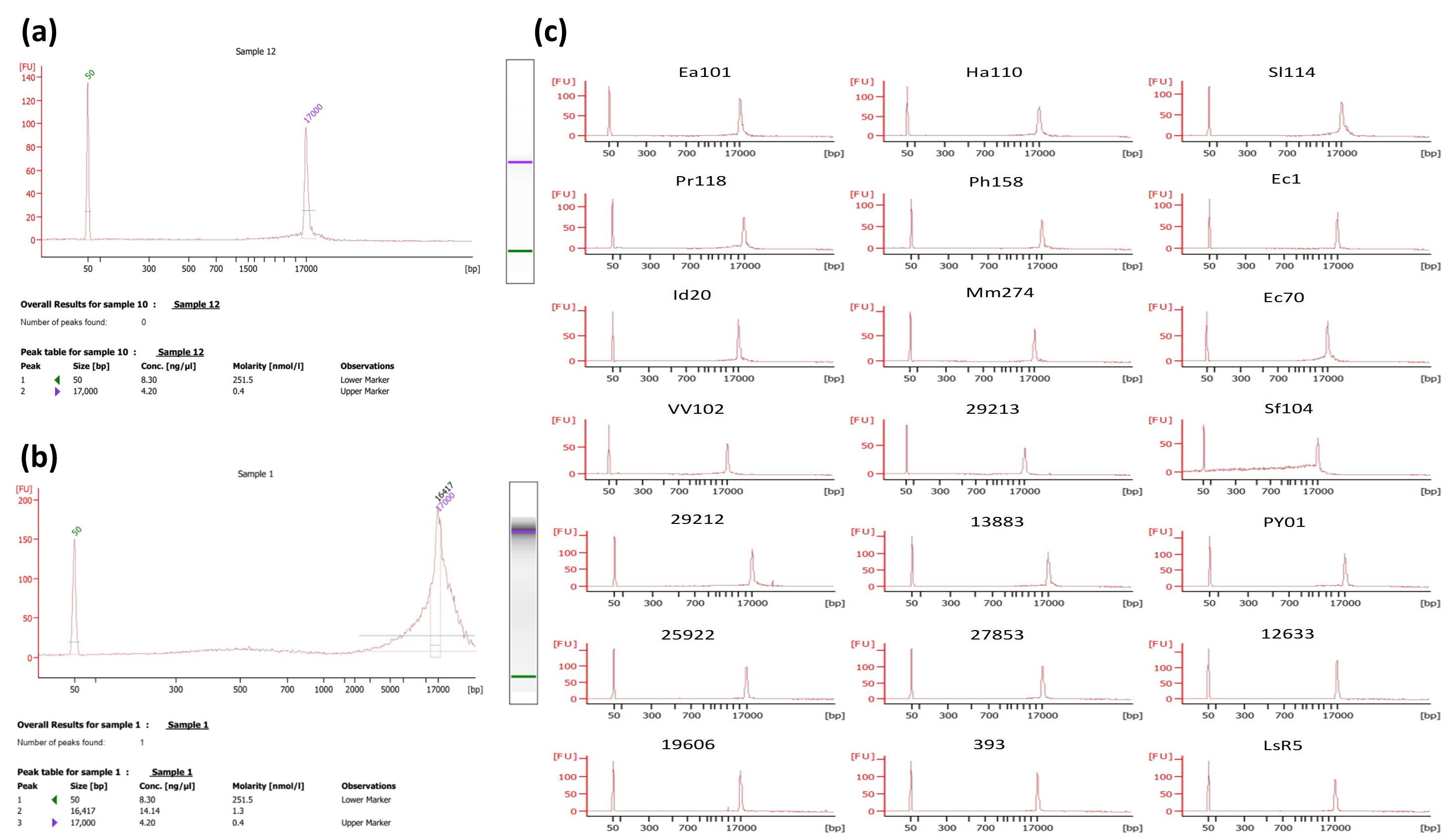


**Supplementary Figure S6. DNA quantity and quality examined by the Agilent 2100 bioanalyzer. DNA from human metagenome, the mock community BMS21 and the 21 individual isolates were included in the figure.**


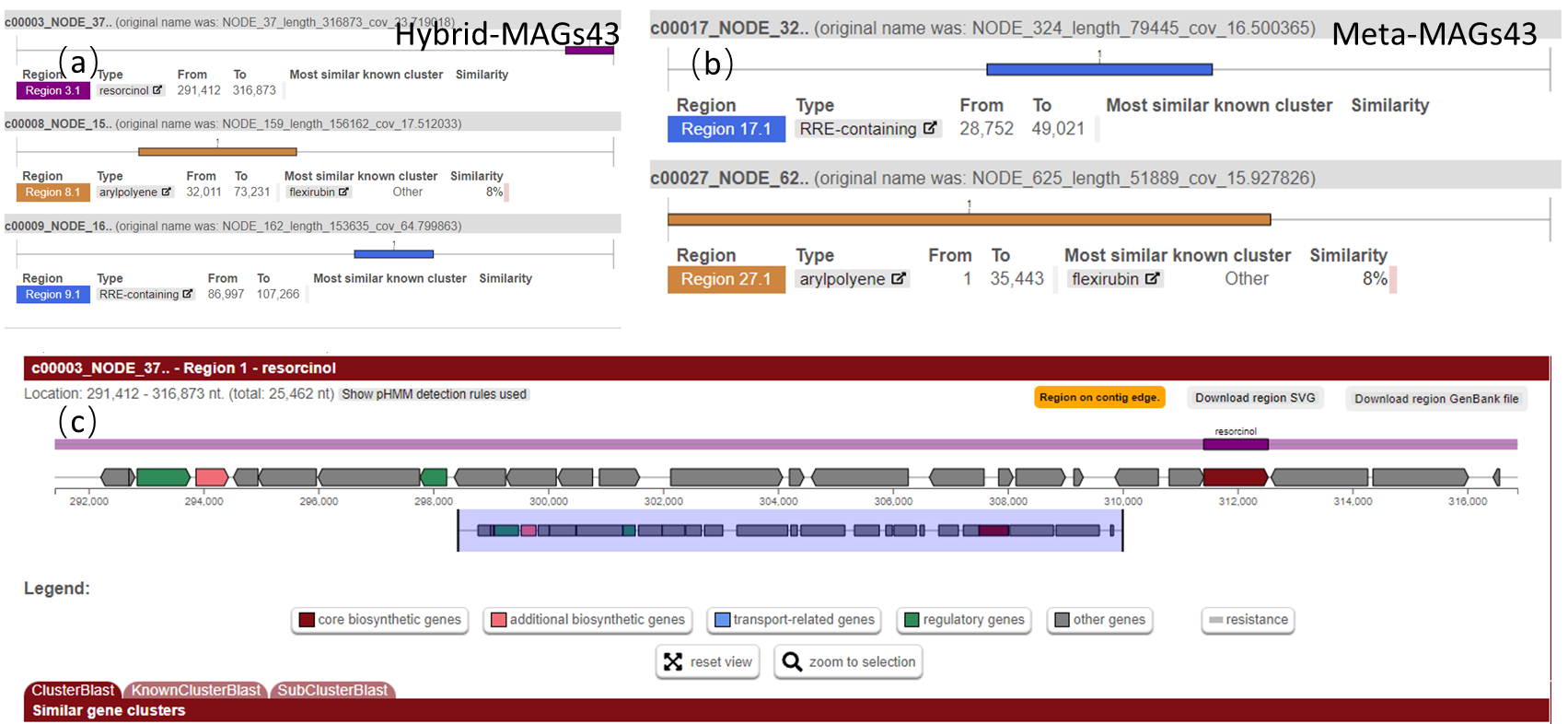


**Supplementary Figure S7 BGS of Hybrid-MAGs43 and Meta-MAGs43.**
